# Mass spectrometry-based metabolomics study of nicotine exposure in THP-1 monocytes

**DOI:** 10.1038/s41598-024-65733-7

**Published:** 2024-06-28

**Authors:** Silvio Uhlig, Bergitte Pearl Olderbø, Jan Tore Samuelsen, Solveig Uvsløkk, Lada Ivanova, Camille Vanderstraeten, Lene Aiko Grutle, Oscar Daniel Rangel-Huerta

**Affiliations:** 1 Nordic Institute of Dental Materials, Sognsveien 70A, 0855 Oslo, Norway; 2 Toxinology Research Group, Norwegian Veterinary Institute, P.O. Box 64, 1431 Ås, Norway; 3 Department of Bioanalysis, Faculty of Pharmaceutical Sciences, Ghent University, Ottergemsesteenweg 460, 9000 Gent, Belgium

**Keywords:** Bioinformatics, Biotransformation, Cell culture, Metabolomics, Nicotine, Metabolomics, Biomarkers, Analytical chemistry, Biochemistry, Chemical biology, Cheminformatics

## Abstract

The tobacco alkaloid nicotine is known for its activation of neuronal nicotinic acetylcholine receptors. Nicotine is consumed in different ways such as through conventional smoking, e-cigarettes, snuff or nicotine pouches. The use of snuff has been associated with several adverse health effects, such as inflammatory reactions of the oral mucosa and oral cavity cancer. We performed a metabolomic analysis of nicotine-exposed THP-1 human monocytes. Cells were exposed to 5 mM of the alkaloid for up to 4 h, and cell extracts and medium subjected to untargeted liquid chromatography high-resolution mass spectrometry. Raw data processing revealed 17 nicotine biotransformation products. Among these, cotinine and nornicotine were identified as the two major cellular biotransformation products. The application of multi- and univariate statistical analyses resulted in the annotation, up to a certain level of identification, of 12 compounds in the cell extracts and 13 compounds in the medium that were altered by nicotine exposure. Of these, four were verified as methylthioadenosine, cytosine, uric acid, and l-glutamate. Methylthioadenosine levels were affected in both cells and the medium, while cytosine, uric acid, and l-glutamate levels were affected in the medium only. The effects of smoking on the pathways involving these metabolites have been previously demonstrated in humans. Most of the other discriminating compounds, which were merely tentatively or not fully identified, were amino acids or amino acid derivatives. In conclusion, our preliminary data suggest that some of the potentially adverse effects related to smoking may also be expected when nicotine is consumed via snuff or nicotine pouches.

## Introduction

**Figure 1:**
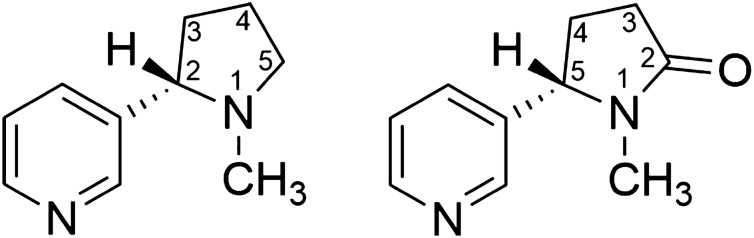
Chemical structures of nicotine (left) and its main biotransformation product cotinine (right).

Nicotine (Fig. 1) is the principal alkaloid in the leaves of the tobacco plant (*Nicotiana* spp.) but is also a natural product in other plants of the Solanaceae family^1,2^. It is a psychoactive substance acting at neuronal nicotinic acetylcholine receptors (nAChRs)^3^. The activation of nAChRs initiates rewarding effects that are the background for the addictive effects of nicotine^3,4^. The metabolism and disposition of nicotine in humans has been studied relatively well, and it has been shown that the small molecule undergoes extensive biotransformation^5^. The traditional form of nicotine consumption is cigarette smoking. Although the role of nicotine for some of the adverse health effects related to smoking of cigarettes is not clear, the compound is the source of carcinogenic nitrosamines that may be formed during tobacco processing or smoking^6^. Nowadays, the use of e-cigarettes is becoming increasingly common, while in some parts of the world nicotine is also frequently consumed using snuff or nicotine pouches^7–9^. The amount of nicotine in a conventional cigarette is similar to that in a nicotine pouch, i.e., in the range between 1 and 10 mg/unit (0.162–1.62 mol/unit)^9,10^. The nicotine release rate from snuff or nicotine pouches is high, and at least 80% of the nicotine is commonly released within 30 min^11,12^. Furthermore, the use of snuff has been related to a number of adverse health effects, such as inflammatory reactions of the oral mucosa and oral cavity cancer^13,14^.

Metabolomics aims to systematically study as many metabolites as possible in a biological system at a given time and may give clues about phenotypic changes related to extrinsic factors such as environmental stressors^15^. The technique has been used a few times to study metabolomic changes related to nicotine exposure. Gómez-Canela et al. used adult zebrafish and aimed to evaluate the potential myotoxicity of nAChR agonists^16^. Other studies focused on the metabolomic changes related to nicotine exposure in bacteria. Ye et al. and Ding et al. investigated the metabolic response of a *Pseoudomonas* sp. and *Escherichia coli*, respectively, to nicotine stress^17,18^, while Chi et al. studied the effects of nicotine on metabolites of the mouse gut microbiome^19^. A few other studies aimed to elucidate metabolomic changes related to tobacco smoke^20–22^. However, as tobacco smoke contains thousands of chemicals any observed effects are likely not directly related to nicotine itself.

The aim of this research was to determine the effects of nicotine on the metabolome of a simple biological model. We selected human monocytes to study effects of short-term exposure to nicotine at a concentration that is likely relevant for the use of snuff or nicotine pouches. The serum nicotine level increases rapidly in humans after exposure to various nicotine delivery systems^23^. Hence, monocytes are being exposed to nicotine in individuals that are users of various nicotine-containing products, like cigarettes and snuff. To make the findings in the current study relevant for a variety of nicotine exposure scenarios, we chose the human-derived monocytic cell line, THP-1, as the model^24^. By providing more insight into metabolic changes induced by nicotine, we could enhance our understanding of the general effects of nicotine in users of snuff and nicotine pouches.

## Materials and methods

### Chemicals and reagents

Water and methanol (MeOH) for liquid chromatography–high resolution mass spectrometry (LC–HRMS) were Optima grade from Fisher Scientific (Oslo, Norway), acetonitrile (MeCN) for LC–HRMS was LC–MS grade from Riedel-de Haën (Honeywell, Germany). RPMI 1640 medium with 2 mM l-glutamine, heat-inactivated foetal bovine serum (FBS), (−)-nicotine (≥ 99%), ammonium carbonate (analytical grade), ammonium acetate (LC–MS Ultra grade) and 25% ammonia solution (LiChropur grade) were from Merck (Merck KGaA, Darmstadt, Germany). Water, MeOH and MeCN for extraction and sample preparation was ULC/MS quality from Biosolve Chimie SARL (Dieuze, France). Methanol calibrant stock solutions (1 mg/mL) of *S*(−)-nicotine for targeted analyses as well as (−)-cotinine, ( ±)-nornicotine and 4-(methylnitrosamino)-1-(3-pyridyl)-1-butanone (nicotine-derived nitrosamine ketone, NNK) were of Cerilliant^®^ quality from Merck. Ammonium formate and formic acid (99%) were ULC/MS quality from Biosolve. HEPES buffer (1 M) in normal saline and sodium pyruvate (100 mM) were from BioWhittaker (Lonza, Basel, Switzerland).

### Cell culture and exposure trial design

THP-1 monocytes were obtained from European Collection of Authenticated Cell Cultures (ECACC, Salisbury, UK) and maintained in RPMI 1640 medium with 2 mM l-glutamine supplemented with 1 mM sodium pyruvate, 10 mM HEPES and 5% FBS. The monocytes were cultured and kept in an exponential growth phase at a density between 0.25 and 1 × 10^6^ cells/mL in 20 mL medium in 75 cm^2^ Falcon cell culture flasks (Corning^®^, VWR International, Radnor, PA, USA) at 37 °C in a humidified incubator in an atmosphere containing 5% CO_2_.

The THP-1 monocytes were seeded in Corning^®^ Costar^®^ TC-treated 6-well flat bottom plates (VWR International) at a density of 0.5 × 10^6^ cells/mL in 2.5 mL medium per well. After resting for 24 h, the cells were exposed to 5 mM nicotine for 1 or 4 h. Stock solutions of nicotine were freshly made in cell culture medium prior to exposure. Quadruplicate samples were collected directly before exposure, as well as following 1 and 4 h of exposure. Control samples, to which only nicotine-free medium was added, were run in parallel. In addition, three solvent control samples and three medium control samples were included that went through the sample preparation.

### Extraction and sample preparation

The extraction and sample preparation procedure was adapted and adjusted from Ser et al.^25^. At the end of the exposure experiment, THP-1 cell suspensions were transferred to 15-mL polypropylene tubes and centrifuged to separate cells and medium (4 °C, 50×*g*). Aliquots (40 µL) of the medium were transferred to 1.5-mL tubes (Sarstedt, Nümbrecht, Germany) and placed on ice, while the remaining medium was discarded. To the cells, 1 mL of 80% MeOH in water was added, which was pre-cooled at − 80 °C for at least 1 h. The tubes were vortexed for ca. 10 s and then incubated on ice for 15 min followed by centrifugation for 10 min (4 °C, 20,000×*g*). The supernatants were transferred to conical glass tubes and evaporated to dryness at 35 °C using a gentle stream of nitrogen. The residue was dissolved in 15 µL of water by vortexing and diluted with 15 µL MeOH/MeCN (1:1). Samples were transferred to chromatography vials with fixed insert and stored at − 80 °C until instrumental analyses.

To the culture medium aliquots (40 µL), 200 µL cold 80% MeOH was added and vortexed for ca. 15 s. The samples were centrifuged for 10 min (4 °C, 20,000×*g*). The supernatants (200 µL) were transferred to chromatography vials with fixed insert and evaporated to dryness at 35 °C using a gentle stream of nitrogen. The dry residue was dissolved in 30 µL of water by vortexing and then diluted with 30 µL of MeOH/MeCN (1:1). Samples were stored at − 80 °C until instrumental analyses.

#### Quality controls

Aliquots (5 µL) of each sample were pooled to obtain a Quality Control (QC) sample, which was used to monitor the system stability through the entire instrumental analysis as well as for data dependent acquisition (DDA).

### Untargeted LC–HRMS and LC–HRMS/MS metabolomics

Samples were randomized and placed into the autosampler tray of the LC–HRMS instrument and kept at 8 °C. The pooled QC sample was run six times at the beginning of the sequence, and every eighth sample throughout the entire LC–HRMS experiment. LC–HRMS analyses were performed using a Q Exactive™ Hybrid Quadrupole-Orbitrap mass spectrometer equipped with a heated electrospray ion source (HESI-II) and coupled to an ultrahigh-performance liquid chromatography (UHPLC) Vanquish Horizon system (Thermo Fisher Scientific, San Jose, CA, USA). Chromatographic separation was achieved by hydrophilic interaction chromatography (HILIC) using a zwitterionic SeQuant ZIC-pHILIC column (Merck; 150 × 4.6 mm, 5 µm). The column was eluted using a mobile phase consisting of 20 mM ammonium carbonate (pH 8.3) and MeCN (B). The elution proceeded isocratically at a constant flow rate of 0.3 mL/min for 1 min with 80% B, followed by a linear gradient to 20% B over 29 min. Finally, the column was flushed with 8% B for 5 min, returned to the initial conditions, and equilibrated for 9 min. The HRMS instrument was run in full-scan positive and negative ion modes using fast polarity switching in the mass-to-charge (*m/z*) range 58 to 870. The HESI-II interface was operated at 300 °C. The spray voltage was 2.8 and 3.2 kV (positive and negative mode, respectively), the ion transfer capillary temperature was 280 °C, the sheath and auxiliary gas flow rates were 35 and 10 units, respectively, and the S-lens RF level was 55%. The automated gain control (AGC) target was set to 5 × 10^5^, and the maximum injection time (IT) was set to 250 ms. A mass resolution of 75,000 full width half maximum (FWHM) at *m/z* 200 was used. All analyses were performed without a lock mass. Xcalibur software (version 4.6) was used for instrument control and LC–HRMS data acquisition.

#### Samples for LC–HRMS/MS data acquisition

In addition to the full-scan analyses, a set of LC–HRMS/MS data was acquired for three different samples: the QC sample, the mixture of nicotine-related metabolites, and a pooled sample of nicotine-exposed samples using DDA. The conditions were as follows: full-scan MS/MS product ion spectra were acquired of the top-five most intense MS ions in the mass range *m/z* 58 to *m/z* 870 with a mass resolution of 17,500 for product ion detection. Fragmentation was performed by applying three different collision energies (NCE 15, 35, and 65) in separate runs independently for each ionization mode. An inclusion list targeting the *m/z* and retention time (± 1 min) for each compound in the in-house library was attached to the method. An exclusion list was generated from the most intense ions present in the first blank and attached to the method to eliminate potential contaminants and increase the number of relevant MS/MS spectra.

#### Processing and quality control of metabolomics data

The raw data from both ionization modes were processed using Compound Discoverer (CD, version 3.3 SP2, Thermo Scientific). First, a retention time alignment was performed with the ChromAlign algorithm^26^ followed by peak picking considering a 5 ppm mass tolerance, a 100,000 minimum peak intensity, and including the following types of ions: [M+H]^+^, [M+NH_4_]^+^, [M+Na]^+^, [M+K]^+^, [M+MeCN+H]^+^, [M+Cl]^−^, [M+MeOH+H]^+^, [M+H−H_2_O]^+^, [M+H−NH_3_]^+^, [M−H]^−^. In the compound-grouping step, the peak area integration was done in extracted ion chromatograms based on the most common ion, with [M+H]^+^ and [M−H]^−^ as preferred ions, and setting the mass tolerance to 5 ppm and retention time tolerance to 0.2 min. Only peaks with an Original Peak Rating above 5 (in a scale of up to 10) in at least 5 samples were considered for further processing. Missing value imputation was performed using the Random Forest Algorithm^27^ considering 100 trees and a maximum number of 10 iterations.

QC correction was performed using the SERRF method^28^, considering 200 trees and samples as one batch, and values were interpolated using non-linear regression. The maximum QC RSD allowed before correction was set to 50%, and the maximum after correction was 25%. The minimum percentage of usable hits (actual signals found) per compound in at least one group was 80%. Samples were normalised to the maximum peak area median of all samples and then was log transformed and Pareto scaled for further statistical processing.

### Statistical analyses

#### Univariate statistical analyses

Paired t-tests were performed between each group at each time point using R^29^.

#### Multivariate statistical analyses

Normalised peak areas acquired in CD were imported into SIMCA (version 16.0.2., Sartorius Stedim Biotech, Umea, Sweden), log-transformed and Pareto scaled prior to multivariate analyses. A principal component analysis (PCA) was performed to detect potential drift and assess data quality. Metabolite patterns and discriminant features were investigated using Orthogonal Partial Least-Squares Discriminant Analysis (OPLS-DA) models. Pair-wise models were created for each group of cell extracts and medium samples, and for each time point independently. Cross-validation of variance (CV-ANOVA) was performed to evaluate the reliability of the models, and p-values ≤ 0.05 were considered significant. The selection of the most significant features for each significant model was determined combining three different cut-off criteria as follows: a p(corr) value ≥|0.50|, a variable importance in the projection (VIP value) ≥ 1.5, as well as a p-value of ≤ 0.05 from the univariate test. The p(corr) value represents the correlation and reliability of the data in Y (exposed or non-exposed for the present study).

### Molecular network analysis

Molecular networking analysis was included within the CD workflow. The parameters were as follows: mass tolerance 2.5 mmu, minimum MSn coverage: 70, minimum fragments: 3, and minimum MSn score: 50. In order to be considered, nodes (compounds) were required to have MSn data and transformation. For exploration of specific nodes, settings were modified dynamically.

### Annotation and identification

#### Putative annotations using spectral libraries and in-silico tools

The annotation process of the most relevant metabolites began during the pre-processing in CD with matching the information of the HRMS/MS data obtained from DDA of three different samples, i.e., a pooled QC of cell extract samples and a QC of medium extract samples, a mixture of nicotine metabolite standards containing nicotine, cotinine, nornicotine and nicotine-derived nitrosamine ketone (NKK). Annotations were attained automatically by matching the measured mass spectra to our in-house library (Table S1), comparing mass accuracies, retention time, and isotope patterns^30^. Spectral similarity scores between measured features and reference metabolites were determined in CD using the HighChem HighRes algorithm with a precursor mass tolerance of 10 ppm. For the identification of the metabolites, we set a spectral similarity cut-off of 85%. The use of public spectral libraries contained in CD was included in the annotation process to provide tentative annotations.

For the manual annotation of the remaining features with unknown identity, the raw data was transformed into the open-source format,*.msp. Then, the data was imported into the automated class assignment and ontology prediction tool CANOPUS^31^ that is an integrative part of the SIRIUS software (v.5.8.3)^32^. Using CANOPUS allowed assigning the chemical fingerprint and the chemical class following the ClassyFire ontology^33^.

### Level of identification

Distinct levels of annotation confidence were assigned to the compounds, following the current guidelines for annotation or identification for metabolomic studies^34^. Level 1 corresponds to the unambiguous identification of a metabolite by matching it to a reference compound with at least two independent and orthogonal properties (e.g., retention time and accurate *m/z*). Level 2 describes the putative identification of metabolites based on the spectral similarities between the HRMS/MS fragmentation data and spectral libraries. Level 3 refers to metabolite classes tentatively characterized by spectral similarity to published HRMS/MS fragmentation data. Level 4 indicates unidentified or unclassified metabolites that are differentiable because of their specific spectral data.

### Solid-phase extraction (SPE) of nicotine-related metabolites from cell extracts using cation exchange

For the semi-targeted extraction of nicotine-related metabolites, THP-1 cell lysates were extracted using cation exchange SPE. THP-1 cell suspensions were transferred to 15-mL polypropylene tubes and centrifuged to separate cells and medium (4 °C, 50×*g*). The cells were washed with 4 mL PBS, centrifuged and the supernatant discarded. Another 300 µL of PBS were added to the cells and the suspension transferred to a 1.5-mL Eppendorf tube. The cells were lysed by application of a sonication probe using an amplitude of 30% (Sonics & Materials, Inc., Newton, CT, USA). An Oasis MCX mixed-mode cation exchange cartridge (60 mg, Waters Corporation, Milford, MA, USA) was conditioned with 2 mL methanol, followed by 2 mL water and 2 mL of 20 mM ammonium formate (pH adjusted to 2.5 using formic acid). An aliquot of the cell lysate (200 µL) was diluted with 800 µL water, and 100 µL of formic acid added. The diluted extract was applied to the column, which was washed with 20 mM ammonium formate in water, followed by 20 mM ammonium formate/methanol (1:4, v/v). The column was dried under vacuum, and nicotine-related metabolites eluted with 1 mL 5% aqueous ammonia solution/methanol (1:9, v/v).

## Results and discussion

### Cell extraction

An initial trial was conducted to evaluate if the evaporation of the THP-1 cell extracts and medium samples could be omitted. Thus, extracts from triplicate exposures with 5 mM nicotine (4 h) were split into two, and half of the extracts evaporated and dissolved as outlined above or analysed without further processing. We found that concentration of the extracts was necessary as otherwise the metabolite coverage was rather low (Fig. S1). This is in-line with other studies that included an evaporation step and applied either a vacuum evaporator or nitrogen-assisted evaporation^25,35^. Another consideration was the inclusion of a washing step of the cells prior to their extraction. Ser et al. reported that, with some exceptions, signal intensities decreased when cells were washed with PBS or water, and thus it was omitted in our study^25^.

### Quality assessment and overview of the LC–HRMS/MS dataset

The metabolomics analyses and pre-processing in Compound Discoverer software resulted in the detection of 1233 compounds, which was reduced to 532 by retaining only those present in at least 80% of the samples from one group. By using a web-based service on automated structural classification of chemical entities, ClassyFire, the 532 compounds were assigned to 69 different chemical classes (Fig. S2). ClassyFire indicated that the highest proportion of the compounds in the cell extracts and medium samples were α-amino acids or derivatives of α-amino acids (Fig. S2). Principal component analysis showed overlapping scores for the QC samples indicating the absence of instrumental drift (Fig. S3). Furthermore, the PCA scores plot including only the study samples showed a clear separation between the cell extract and medium samples from the nicotine-exposed and non-exposed cells (Figs. S4–S6).

### Screening for nicotine-related biotransformation products

**Figure 2:**
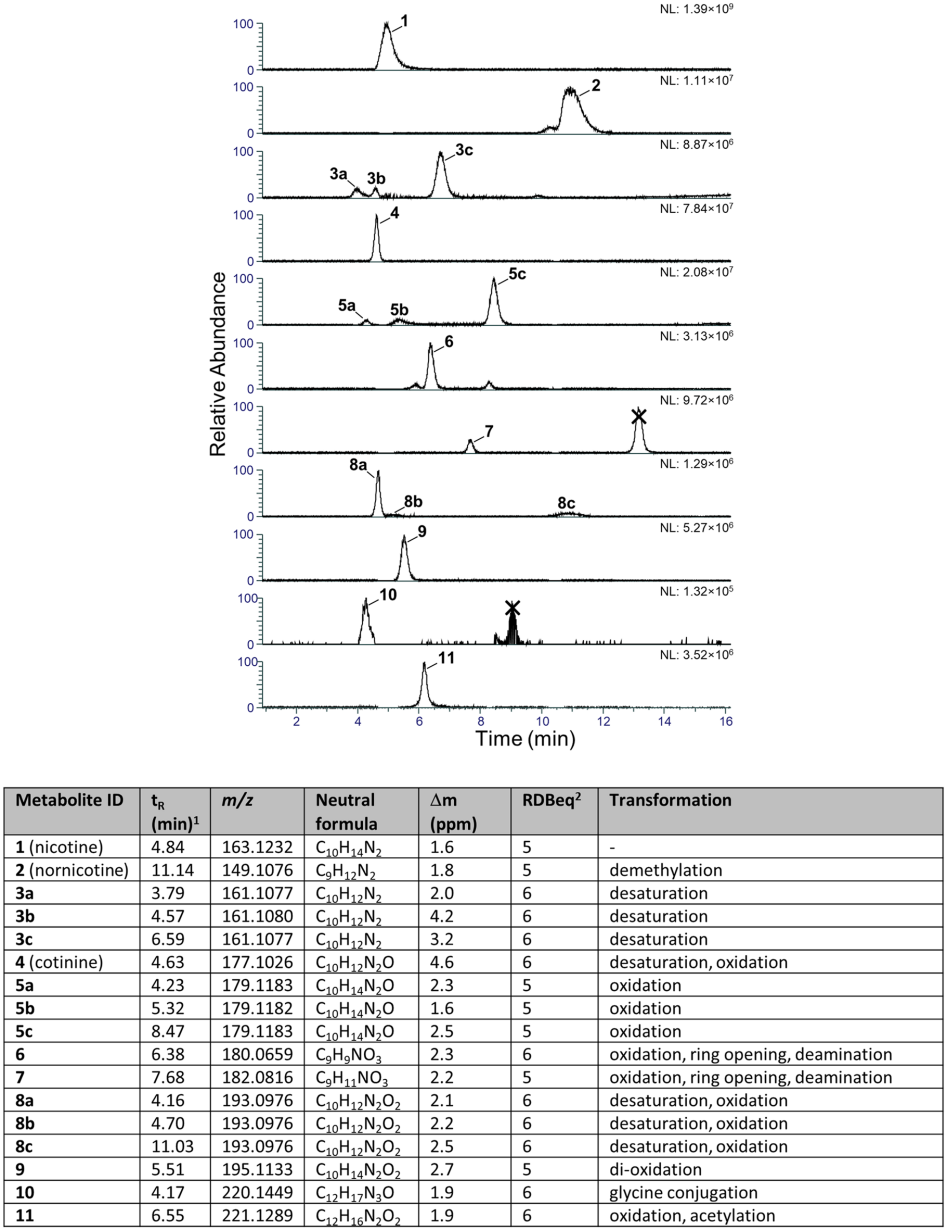
Extracted ion chromatograms (± 4 ppm, except **3** and **4**, which are ± 6 ppm) of the protonated molecules for nicotine and nicotine metabolites in THP-1 cell extracts, as well as LC–HRMS characteristics of verified and putative nicotine biotransformation products. Normalization levels (NL) (arbitrary units) for the chromatograms are indicated in the panel for each type of metabolite to compare individual peak heights. Crossed-out peaks are from compounds that were present in control extracts in comparable peak intensity.

**Figure 3:**
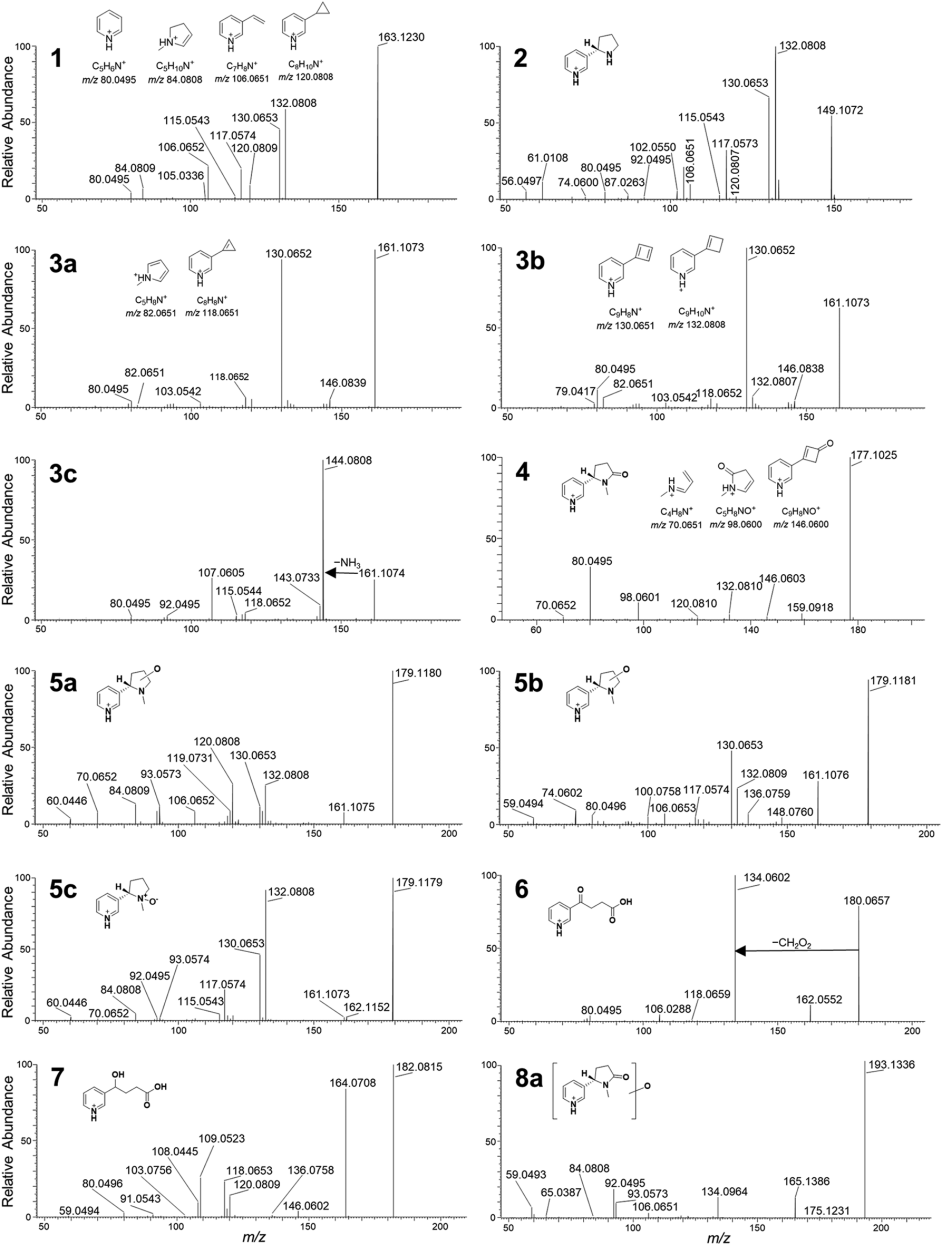

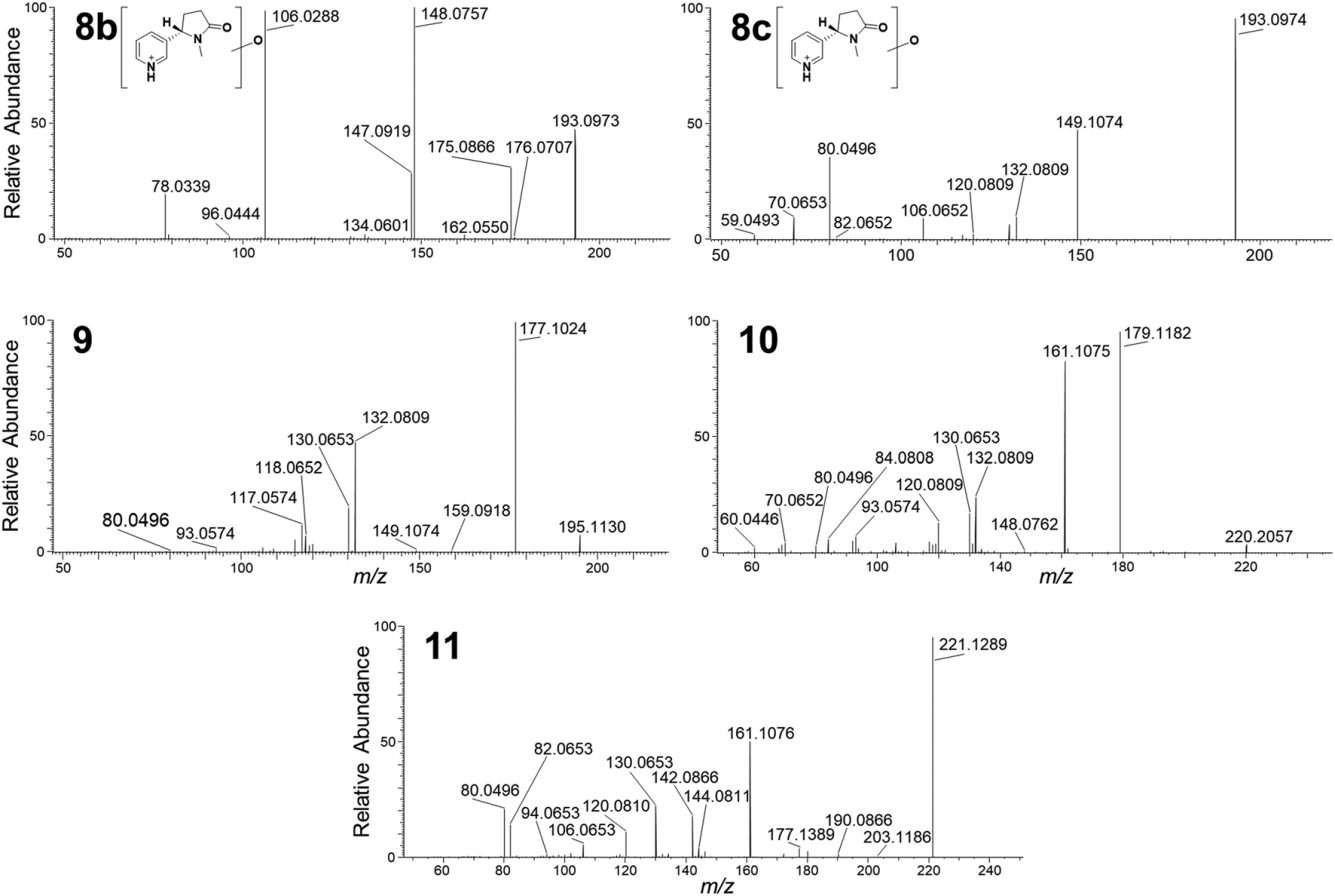
Product ion spectra from HRMS/MS of the protonated molecules of nicotine (**1**) and identified as well as putatively annotated biotransformation products in THP-1 cells, including tentative structures of nicotine metabolites (in bold) and plausible structures of diagnostic product ions.

Nicotine-related metabolites were extracted from the dataset in multiple ways. We used the built-in biotransformation prediction option in Compound Discoverer software as follows: (i) the nicotine structure was assigned as parent compound, and a list of theoretical biotransformation products, including their expected *m/z* was generated; (ii) during processing, a list of expected compounds was generated based on that list, and (iii) MS/MS fragmentation spectra (if available) of expected compounds were compared with spectra of the parent compound and annotation of matching fragment structures and biotransformation-shifted fragments was obtained and scored. In addition, we also used the molecular networking function of the software (Fig. S7) and combined the information obtained from the network with the list of expected compounds. The analyses resulted in a list of 17 putative biotransformation products, including cotinine and nornicotine that were verified by comparison to reference standards (Fig. 2). The observed biotransformation products were in most cases the result of oxidation, desaturation, demethylation and deamination reactions, some of which lead to opening of the pyrrolidyl-ring and production of putative keto- and hydroxy acids (metabolites **6** and **7**) (Fig. 3)^5,36^. As far as can be assessed from relative peak areas of the protonated molecules, cotinine (**4**) was the major metabolite in THP-1 cells. Other major metabolites were nornicotine (**2**) and oxidation product **5c** (Fig. 3). Oxidation product **5c** was chromatographically well separated from the other two oxidation products, **5a** and **5b**, which eluted at a similar retention time as nicotine itself. Cytochrome P450-mediated hydroxylation of nicotine has been reported to occur at the pyrrolidyl-carbon-2, in addition to *N*-oxidation of the pyrrolidone-nitrogen, while cotinine may also be hydroxylated in the 3-position^5^. Although the HRMS/MS spectra were not informative with regard to the site of hydroxylation, the spectra of **5c** showed loss of a hydroxyl radical (*m/z* 162.1152, Δ_m_ = 1.1 ppm) (Fig. 3). This is a characteristic fragmentation pathway of *N*-oxides^37^, and thus it is likely that **5c** is nicotine-*N*-oxide. The elemental compositions of the three oxidation products **8a**–**8c** suggest oxidation products of cotinine (− H_2_, + O_2_ relative to nicotine). Hydroxylation of cotinine has been reported in the 3- and 5-position as well as the *N*-methyl group, and in addition the pyridyl-nitrogen may become oxidised and give rise to another *N*-oxide^5^. The HRMS/MS spectra gave no reliable information on where in the molecule the oxidation in **8a**–**8c** occurred (Fig. 3). The product ion spectra of **6** and **7** both well supported the presence of a carboxylic acid group in their molecules. This was especially prominent in the spectrum of **6**, which showed a base peak (*m/z* 134.0602) that corresponded to the neutral loss of formic acid (Fig. 3). Metabolites **10** and **11** represent more rare biotransformation products, and could be due to glycine conjugation, and oxidation/acetylation, respectively. Although **10** was of rather low peak intensity, the HRMS/MS spectra showed that both were nicotine-related compounds. Acetylation of e.g., a hydroxyl group in **11** was supported by the neutral loss of acetic acid giving rise to the *m/z* 161.1076 product ion (Fig. 3). We also developed a cation-exchange SPE procedure for semi-targeted extraction of nicotine-related compounds. The procedure was optimised using nicotine, cotinine, and nornicotine as reference standards (data not shown). To increase the specificity of the extraction, we used a sonication probe to lyse the cells in PBS. This approach enabled the detection of metabolites **2**, **3a**–**c**, **4**, **5b**-**c**, **6** and **8a**, further supporting that these were nicotine-related metabolites (Fig. S8).

The detection of nicotine biotransformation products raises the question of their contribution to any of the observed biological effects. Both cotinine and nornicotine have been reported to possess neuropharmacological activity^38–40^, which is however less relevant for this study. Cotinine has been reported to have a stronger effect on lipid peroxidation in the hippocampus than nicotine^38^. It also affected ERK 1/2 phosphorylation and antioxidant capacity to the same extend as nicotine^38^. These data suggest that some nicotine metabolites could have similar effects in THP-1 cells, as described below.

### Determination of differential metabolites

Orthogonal PLS-DA was carried out to infer metabolites that were present in higher or lower concentration in cell extracts or medium samples following nicotine exposure, relative to unexposed controls. Individual models were assessed comparing both the cell extracts and the medium samples after 1 h (t1) and 4 h (t4) of exposure (Table 1, Figs. S9–S12). Cross-validation ANOVA showed that the OPLS-DA model from the cell extracts at t4 was not significant (Table 1). After the exploration of the significant models, a list of discriminant features was extracted (Supplementary Material) for each model, using the inclusion criteria described in the Materials and Methods. Since nicotine-related biotransformation products constituted a relatively large weight in the models, a set of new models was created to investigate potential metabolic differences between samples (Table 2). This had only a minor effect on the model characteristics, and likewise did not change the fact that the model based on the cell extract data at t4 was not significant per CV-ANOVA. The new list of differential metabolites was filtered against an in-house library of 505 unique metabolites (Table S1), that had previously been tested and optimised using the HILIC–HRMS/MS method used in this study. This revealed four metabolites that were affected in THP-1 by exposure with nicotine. These were, in the order of decreasing VIP scores, cytosine, methylthioadenosine, uric acid and l-glutamate (Table 3, Figs. S13 and S14). Methylthioadenosine was the only identified metabolite that was detected at significantly different (i.e., significantly lower) concentrations in the cell extract following nicotine exposure. Its concentration changed also significantly in the culture medium at t1 (Table 3).

**Table 1:** Summary of OPLS-DA model parameters from LC–HRMS metabolomics data of cell extracts and medium following exposure to nicotine versus control.

Components	R2X(cum)	R2Y(cum)	Q2(cum)	Comparison	CV-ANOVA *P*-value
1 + 2	0.825	0.998	0.964	Cell extract, exposed (1 h) versus control	0.017
1 + 1	0.69	0.960	0.561	Cell extract, exposed (4 h) versus control	Not significant
1 + 2	0.638	1	0.954	Medium extract, exposed (1 h) versus control	0.024
1 + 1	0.469	0.989	0.869	Medium extract, exposed (4 h) versus control	0.030

**Table 2:** Summary of OPLS-DA model parameters from LC–HRMS metabolomics data of cell extracts and medium following exposure to nicotine versus control, excluding nicotine-related compounds.

Components	R2X(cum)	R2Y(cum)	Q2(cum)	Comparison	CV-ANOVA *P*-value
1 + 2	0.82	0.997	0.942	Cell extract, exposed (1 h) versus control	0.03
1 + 1	0.68	0.930	0.441	Cell extract, exposed (4 h) versus control	Not significant
1 + 1	0.783	0.999	0.990	Medium extract, exposed (1 h) versus control	0.0001
1 + 1	0.444	0.990	0.849	Medium extract, exposed (4 h) versus control	0.04

**Table 3:** Differential metabolites that were verified using an in-house database that was generated using authentic standards.

Metabolite	Model	Formula^a^	Ion	*m/z*	t_R_^b^ (min)	VIP^c^	*P*-value^d^	Direction^e^
Methylthioadenosine	Cell extract, exposed versus control (1 h)	C_11_H_15_N_5_O_3_S	[M+H]^+^	298.097	6.55	2.02	1.04 × 10^−6^	↓
l-glutamate	Medium extract, exposed (1 h) versus control	C_5_H_9_NO_4_	[M+H]^+^	146.046	15.34	1.83	0.01	↑
Methylthioadenosine		C_11_H_15_N_5_O_3_S	[M+H]^+^	298.097	6.55	1.74	0.02	↓
Cytosine	Medium extract, exposed (4 h) versus control	C_4_H_5_N_3_O	[M+H]^+^	112.051	10.35	2.27	0.01	↓
Uric acid		C_5_H_4_N_4_O_3_	[M−H]^−^	167.021	13.76	1.92	0.02	↑

^a^Neutral formula.

^b^t_R_: retention time.

^c^Variable importance in projection score from OPLS-DA model.

^d^*P*-value from pairwise *t*-test.

^e^Relative change in concentration following exposure with nicotine, i.e., increase (↑) or decrease (↓).

The metabolites were selected from OPLS-DA models based on a VIP score ≥ 1.5 and additional filtering using a *P*-value of ≤ 0.05 from a paired *t*-test.

**Figure 4:**
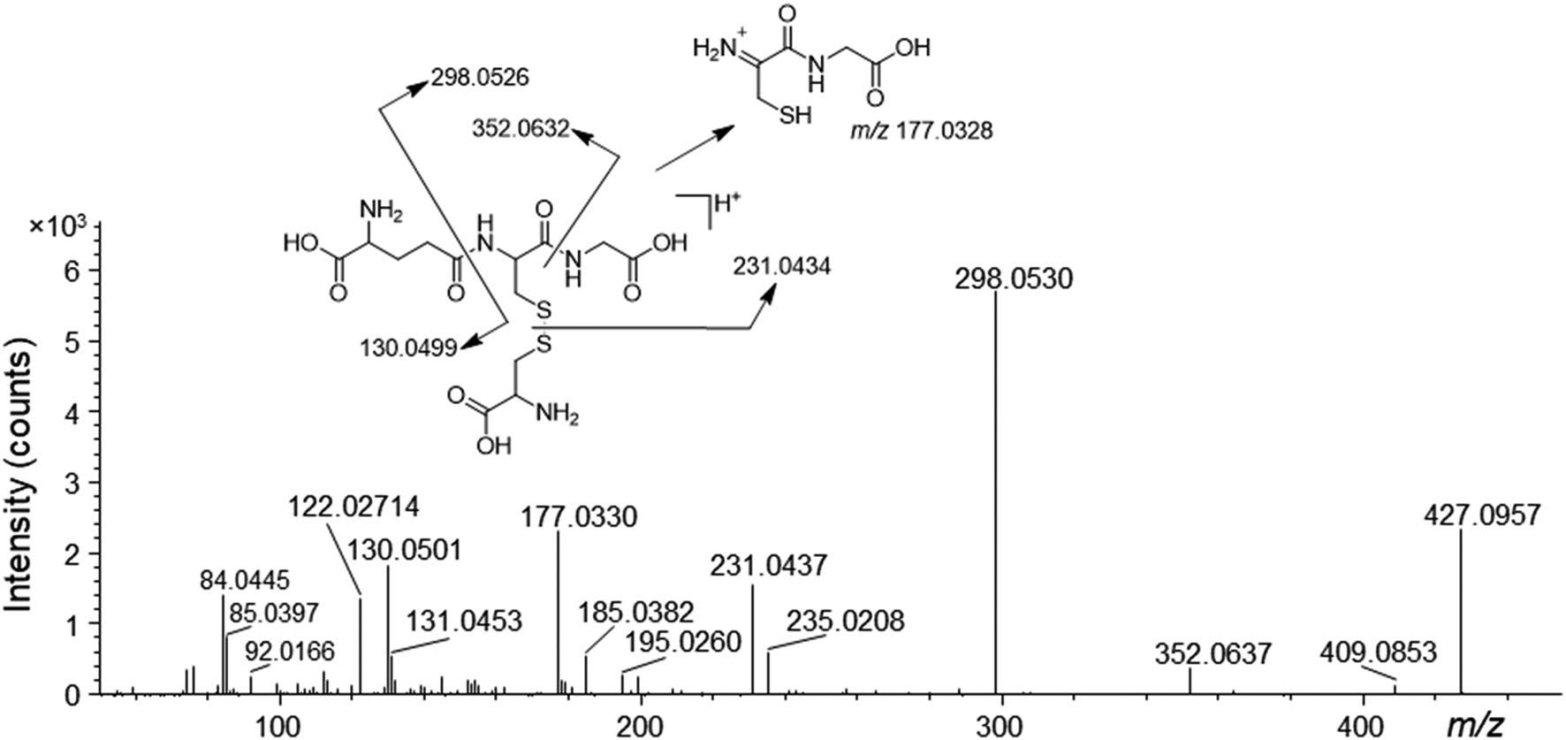
Product ion spectra from HRMS/MS of the protonated molecules of putative glutathionyl-cysteine, including explanations for possible fragmentation pathways.

Efforts to obtain tentative annotations for the remaining metabolites included application of the mzCloud mass spectral database as well as the compound class annotation tool CANOPUS (Tables S2–S4)^31^. This approach resulted in the annotation, up to a certain level of identification, of 12 differential compounds in the cell extracts and 13 differential compounds in the medium (Tables 4 and 5). Furthermore, there was only one metabolite that met the inclusion criteria in the t1 medium samples. The majority of the differential compounds in the cell extracts were amino acids or amino acid derivatives (Table 4). Of the 12 metabolites, three were putatively identified at the compound level with plausible annotations, i.e., maleamic acid, *S*-glutathionyl-l-cysteine and *N*-acetylneuraminic acid. However, the annotation of the *m/z* 114.019 compound as maleamic acid was based on its MS1 characteristics and a MS/MS spectrum of relatively low diagnostic value (only one major product ion) (data not shown). Furthermore, its ClassyFire annotation from CANOPUS as α-amino acid or derivative thereof had a relatively low probability score (Table 4). The tentative annotation of *S*-glutathionyl-l-cysteine was well supported by the presence of diagnostic product ions and high probability score from ClassyFire (Fig. 4 and Table 4). Comparison of the HRMS/MS spectrum of an authentic standard of *N*-acetylneuraminic acid with that of the putatively annotated compound in THP-1 showed that their identities did not match.

**Table 4:** Differential metabolites in THP-1 cell extracts (1 h exposure) for which no reference standards were available.

Formula^a^	*m/z*	Ion	Δm (ppm)	t_R_^b^ (min)	VIP^c^	*P*-value^d^	Putative annotation	Most specific class^e^	Probability	Class or subclass^f^	Direction^g^
C_4_H_5_NO_3_	114.019	[M−H]^−^	− 3.7	15.55	2.22	< 0.01	Maleamic acid	α-amino acids and derivatives	0.54	Carboxylic acids and derivatives	↓
C_13_H_22_N_4_O_8_S_2_	427.096	[M+H]^+^	0.39	17.29	2.13	0.03	*S*-glutathionyl-l-cysteine	γ-glutamyl peptides	0.99	–	↓
C_7_H_14_O_8_	225.061	[M−H]^−^	− 1.9	17.70	2.06	< 0.01	–	Quinic acids and derivatives	0.54	Cyclic alcohols and derivatives	↑
C_6_H_9_NO_4_S	190.018	[M−H]^−^	− 0.79	16.63	1.99	< 0.01	–	α-amino acids	0.62	Amino acids and derivatives	↓
C_30_H_50_O_2_	460.415	[M+NH_4_]^+^	0.96	3.12	1.86	< 0.01	–	Prenol lipids	0.80	Lipids and lipid-like molecules	↑
C_11_H_21_NO_9_	310.114	[M−H]^−^	− 1.5	14.73	1.86	< 0.01	–	Fatty acyl glycosides of mono- and disaccharides	0.57	–	↑
C_6_H_10_O_4_S	177.022	[M−H]^−^	− 2.5	7.66	1.75	< 0.01	–	Short-chain hydroxy acids and derivatives	0.67	Organic acids and derivatives	↑
C_13_H_29_N_3_O_6_S	356.185	[M+H]^+^	0.47	11.09	1.64	0.03	–	Amino acids and derivatives	0.84	Organic acids and derivatives	↓
C_13_H_24_N_2_O_7_S	351.122	[M−H]^−^	− 2.0	8.75	1.58	0.02	–	β-amino acids and derivatives	0.76	Carboxylic acids and derivatives	↓
C_34_H_44_N_5_O_3_P	602.323	[M+H]^+^	− 4.1	3.87	1.54	0.02	–	Amino acids and derivatives	0.80	Carboxylic acids and derivatives	↓
C_11_H_16_N_2_O_2_	209.129	[M+H]^+^	1.2	8.72	1.53	0.05	–	α-amino acids and derivatives	0.86	Amino acids and derivatives	↑
C_11_H_19_NO_9_	310.114	[M+H]^+^	0.17	16.89	1.52	0.05	*N*-acetylneuraminic acid^h^	*N*-acylneuraminic acids	0.81	Sugar acids and derivatives	↑

The table includes only metabolites for which some level of structural information could be obtained. Inclusion criteria were VIP ≥ 1.5, *P* ≤ 0.05 and available MS/MS data.

^a^Neutral formula from Compound Discoverer software.

^b^t_R_: retention time.

^c^Variable importance in projection score from OPLS-DA model.

^d^*P*-value from pairwise *t*-test.

^e^ClassyFire most specific compound class from CANOPUS.

^f^ClassyFire level 5, subclass, class or superclass with probability ≥ 0.85.

^g^Relative change in concentration following exposure with nicotine, i.e., increase (↑) or decrease (↓).

^h^Comparison of the HRMS/MS spectrum with that of a *N*-acetylneuraminic acid reference standard showed no match.

**Table 5:** Differential metabolites in THP-1 medium for which no reference standards were available.

Formula^a^	*m/z*	Ion	Δm (ppm)	t_R_^b^ (min)	VIP^c^	*P*-value^d^	Putative annotation	Most specific class^e^	Probability	Class or subclass^f^	Direction^g^
C_9_H_11_NO_3_^h^	182.081	[M+H]^+^	1.4	8.10	2.18	< 0.01	–	Aryl alkyl ketones	0.96	–	↑
C_9_H_9_N^i^	132.081	[M+H]^+^	1.0	4.79	2.31	< 0.01	Methylindole	Indoles	0.77	Organoheterocyclic Compounds	↑
C_5_H_10_N_2_O_2_^i^	147.077	[M+H]^+^	1.9	11.44	2.27	< 0.01	Ureidoisobutyric acid	α-amino acids	0.95		↑
C_4_H_10_N_2_O_2_S^i^	151.054	[M+H]^+^	2.0	13.71	2.23	0.03	–	Monoalkylamines	0.62	Organonitrogen compounds	↑
C_6_H_6_O_4_S^i^	172.991	[M−H]^−^	− 3.1	4.85	2.16	< 0.01	–	Phenylsulfates	0.68	Organic sulfuric acids and derivatives	↓
C_4_H_7_NO_2_S^i^	134.027	[M+H]^+^	− 0.11	8.75	2.10	0.03	–	α-amino acids	0.59	Amino acids and derivatives	↑
C_9_H_13_N_3_O_4_^i^	228.098	[M+H]^+^	1.3	10.34	2.05	< 0.01	deoxycytidine	Pyrimidine 2ʹ-deoxyribonucleosides	> 0.99		↓
C_40_H_78_NO_8_P^i^	732.555	[M+H]^+^	1.3	3.25	1.99	< 0.01	PE-Me(16:0/18:1)	Phosphatidylcholines	0.99		↓
C_5_H_11_ClN_2_O_3_^i^	181.038	[M−H]^−^	− 2.7	15.35	1.82	0.03	–	α-amino acids	0.53	Amino acids and derivatives	↑
C_9_H_7_N^i^	130.065	[M+H]^+^	1.3	4.75	1.77	0.02	–	Benzenoids	0.97		↑
C_10_H_20_N_2_O_5_^i^	249.145	[M+H]^+^	1.1	7.88	1.74	< 0.01	–	α-amino acids and derivatives	0.77	amino acids and Derivatives	↑
C_10_H_20_O_11_^i^	315.093	[M−H]^−^	− 1.5	13.69	1.66	< 0.01	–	Fatty acyl glycosides of mono- and disaccharides	0.51	–	↑

The table includes only metabolites for which some level of structural information could be obtained. Inclusion criteria were VIP ≥ 1.5, *P* ≤ 0.05 and available MS/MS data.

^a^Neutral formula from Compound Discoverer software.

^b^t_R_: retention time.

^c^Variable importance in projection score from OPLS-DA model.

^d^*P*-value from pairwise *t*-test.

^e^ClassyFire most specific compound class from CANOPUS.

^f^ClassyFire level 5, subclass, class or superclass with probability ≥ 0.85.

^g^Relative change in concentration following exposure with nicotine, i.e., increase (↑) or decrease (↓).

^h^1 h exposure.

^i^4 h exposure.

### Discussion of the observed effects of nicotine on the THP-1 metabolome

#### Cytosine and uric acid

Cytosine is a pyrimidine derivative and one of the four bases that are found in DNA and RNA. Exposure of THP-1 cells to nicotine resulted in a marked relative decrease in cytosine intracellularly, as well as in the medium (Fig. S15). The difference between exposed cells and controls increased with time but was statistically significant (by means of both univariate and multivariate statistics) only in the medium after 4 h of exposure (Table 3, Fig. S15). A possible explanation of the observed decrease in cytosine levels could be methylation of DNA. This is an important type of DNA modification and is connected to many pathologies and diseases^41^. It commonly occurs at C-5 of cytosine, and 5-methylcytosine has been shown to be a valuable marker for DNA methylation^42^. Cigarette smoking modifies the methylation pattern of DNA via different mechanisms, although the role of nicotine is less clear^43^. We were not able to detect 5-methylcytosine, which was part of our in-house library of reference standards. Since 5-methylcytosine may be further oxidized^44^, we also did a targeted search for putative hydroxymethylcytosine in the raw data. Furthermore, we looked for cytosine-related compounds using molecular networking, but without success. We were thus not able to support the hypothesis that the observed decrease in cytosine levels could be related to DNA methylation. Another possibility is that the reduced cytosine levels could be the result of increased cytosine catabolism. In bacteria, nicotine stress up to a certain amount of nicotine resulted in an increase of nucleoside biosynthesis^17,18^. In contrast, a significant depletion of pyrimidine was observed ^17,18^. Catabolism of pyrimidines results in the end products carbon dioxide and ammonia. Uric acid is the final breakdown product of purine base catabolism. Compared to the control (both cells and medium), our findings suggest a marked increase in purine breakdown in nicotine-exposed THP-1 cells (Table 3, Fig. S16). Uric acid levels increased significantly in the microbiome of male mice that were orally administered with nicotine^19^. Elevated serum uric acid levels have previously been indicated in smokers^45,46^. Although the underlying mechanism of a possible link between smoking and serum uric acid levels remains unclear, our data indicate a link to the nicotine component of cigarette smoke. However, we were not able to detect purine bases in our samples. Taken together, the findings could point towards a generally increased nucleotide catabolism after nicotine exposure. Studies focusing more on detailed mechanisms are necessary to further shed light on a possible link between nicotine exposure and nucleotide metabolism.

#### 5ʹ-(Methylthio)-5ʹ-deoxyadenosine (MTA)

MTA is primarily formed from *S*-adenosylmethionine (SAM) during synthesis of the polyamines spermine and spermidine^47^. SAM levels in THP-1 cells were not significantly affected during the 4-h exposure to nicotine. Furthermore, we did not observe an effect of nicotine exposure on spermidine levels, whereas spermine was not among the reference metabolites of our in-house library. However, none of the metabolites that differentiated nicotine-exposed THP-1 cells or media from controls matched the mass spectrometric characteristics of spermine. An increase in MTA as a result of nicotine exposure has previously been shown in the alveolar regions of the lungs of rats^48^. In that study, rats were infused with nicotine over a period of three weeks. The general effect of long-term exposure was increased polyamine metabolism, primarily manifested by SAM depletion and upregulation of ornithine decarboxylase^49^. The depletion of SAM in the study by Moncada et al. was associated with an increase in MTA and decarboxylated SAM. However, MTA was downregulated during the short-term exposure of THP-1 cells with nicotine in our study with significant impact on the intracellular levels, but also in the culture medium (Table 3, Fig. S17). The trend to relatively lower MTA levels in the nicotine-exposed group was also evident after 4 h of exposure, but the difference was not statistically significant (Fig. S17). Thus, our findings also indicate a short-term effect of nicotine on the polyamine biosynthetic pathway and/or methionine salvage pathway. It should be noted that metabolism of MTA is facilitated by 5′-MTA phosphorylase (MTAP)^49^. Thus, the decrease of MTA in THP-1 cells in our study could also be caused by an induction effect of nicotine on MTAP. MTA was recently identified as a sputum biomarker in smokers with chronic obstructive lung disease (COPD) in comparison to healthy nonsmokers^50^. The authors explained the observation by an effect of smoking on the methionine salvage pathway rather than a direct effect on polyamine metabolism. Finally, significantly reduced putrescine levels have been reported in *Pseudomonas* sp. HF-1 following nicotine exposure for 18 h^18^.

#### *l*-glutamate

l-glutamate is an important non-essential amino acid involved in many cellular processes, such as interconversion to α-ketoglutarate in the citric acid cycle for generating cellular energy, functioning as an excitatory neurotransmitter in the central nervous system or for synthesis of the antioxidant glutathione^51^. Changes in l-glutamate concentration after smoking in metabolomics studies have previously been observed^52,53^. The observed increase of l-glutamate in the cell culture medium of the nicotine-exposed group in our study (Table 3, Fig. S18) is in-line with the observation by Harada et al. The authors reported significantly increased plasma glutamate levels in blood from smokers. In contrast, Hsu et al. observed lower plasma glutamate concentrations in cigarette smokers directly after smoking. Furthermore, glutamate levels decreased in bacterial *Pseudomonas* sp. and *E. coli* models that were treated with nicotine^17,18^, while the glutamate levels in the gut microbiome of both male and female mice increased after oral nicotine administration^19^. It appears that there are contradictory outcomes in which direction the l-glutamate concentration changes, however, a consistent observation is the altered l-glutamate levels after exposure. The observed relative increase in concentration in the medium in our study following nicotine exposure for 1 h could be explained by a potential secretion of l-glutamate from the exposed THP-1 cells into the surrounding medium. A trend to elevated l-glutamate levels in the nicotine-exposed group was also evident after 4 h, but it was not statistically significant (Fig. S18). An increased l-glutamate secretion was observed by others when monocytic THP-1 cells differentiate into macrophages following treatment with inflammatory mediators such as phorbol myristate acetate (PMA) or zymosan^54^. This suggests that nicotine may similarly influence immune responses thereby resulting in l-glutamate secretion. Glutamate may be secreted into the medium through the l-cystine/l-glutamate transport system in exchange for extracellular cystine^55^. Cystine is in a dynamic equilibrium with cysteine. Intracellularly, cystine is rapidly reduced to cysteine and subsequently utilized for the biosynthesis of peptides such as glutathione, as well as proteins. This exchange between glutamate and cystine normally occurs in a 1:1 ratio. The THP-1 cells may therefore increase their glutathione synthesis in response to nicotine exposure. An effect of nicotine on cysteine levels (observed as cystine due to fast autoxidation in the extracts) or glutathione has not been observed in our study. It should be noted that the intracellular levels of a metabolite that was tentatively identified as *S*-glutathionyl-l-cysteine were significantly decreased in the nicotine exposed group after 1 h of exposure (Table 4, Fig. 4), but a possible link to the above-mentioned pathways could not be established.

#### Effects on amino acid ratios

Both the absolute and relative levels of several amino acids were significantly affected by nicotine exposure (Tables S5 and S6). Short-term disturbance of the urea cycle following nicotine exposure was indicated by significant effects on the proline-to-citrulline and arginine-to-ornithine ratio^56,57^. This was detected both in the cell extracts and medium samples in case of the arginine-to-ornithine ratio, whereas the effect on the proline-to-citrulline ratio was only detected in the medium.

## Conclusions

Simple cell models, such as human THP-1 monocytes, are well suited for the exploration of biochemical events that are related to exposure to bioactive exogenous chemicals. We showed the effects of nicotine exposure on the levels of specific metabolites. These point to potential nicotine-related perturbations in polyamine biosynthesis and/or methionine salvation, increased DNA methylation and/or nucleotide metabolism, as well as pathways that include glutamate. Cigarette smoking has been shown to affect the mentioned pathways in humans. Hence, our findings suggest a link between these bioactivities and the nicotine component of cigarette smoke. This indicates also that other nicotine-containing products, such as nicotine replacement products advertised as “healthy alternatives,” are not risk-free. As a next step, our findings should be validated and studied in more detail using targeted analyses of the affected metabolites that have been identified in this study, including relevant key metabolites of the pathways they are involved in.

### Supplementary Information

Supplementary Information 1.

Supplementary Information 2.

## Data Availability

All data generated in this work are provided within the manuscript or supplementary information files.
