# Supplementary material for: Mass spectrometry-based metabolomics study of nicotine exposure in THP-1 monocytes
Source: Sci Rep. 2024 Jun 28;14:14957. doi: 10.1038/s41598-024-65733-7 (PMC11213872; doi:10.1038/s41598-024-65733-7)
Supplement: Supplementary file 2 — Supplementary Information 2. [file 41598_2024_65733_MOESM2_ESM.docx]

**SUPPLEMENTARY MATERIALS**

**Mass spectrometry-based metabolomics study of nicotine exposure in THP-1 monocytes**

**Silvio Uhlig^1^, Bergitte Pearl Olderbø^1^, Jan Tore Samuelsen^1^, Solveig Uvsløkk^1^, Lada Ivanova^2^, Camille Vanderstraeten^1,3^, Lene Aiko Grutle^1^, Oscar Daniel Rangel-Huerta^2^**

^1^ Nordic Institute of Dental Materials, Sognsveien 70A, 0855 Oslo, Norway

^2^ Toxinology Research Group, Norwegian Veterinary Institute, P.O. Box 64, 1431 Ås, Norway

^3^ Department of Bioanalysis, Faculty of Pharmaceutical Sciences, Ghent University, Ottergemsesteenweg 460, 9000 Gent, Belgium

|  | **Page** |
| --- | --- |
| Fig. S1: Hierarchical clustering analysis of raw data from trial to test the necessity of up-concentration of cell extracts and medium samples | S3 |
| Fig. S2: Chemical class assignment of THP-1 metabolites from ClassyFire | S4 |
| Fig. S3: PCA scores plot for the whole dataset including blanks and quality control samples | S5 |
| Fig. S4: PCA scores plot for the whole dataset excluding blanks and quality control samples | S5 |
| Fig. S5: PCA scores plot for medium samples only | S5 |
| Fig. S6: PCA scores plot for cell extracts only | S6 |
| Fig. S7: OPLS-DA scores plot for cell extract samples, exposure time 1 h | S7 |
| Fig. S8: OPLS-DA scores plot for cell extract samples, exposure time 4 h | S7 |
| Fig. S9: OPLS-DA scores plot for medium samples, exposure time 1 h | S8 |
| Fig. S10: OPLS-DA scores plot for medium samples, exposure time 4 h | S8 |
| Fig. S11: Example of a nicotine molecular network from Compound Discoverer software | S9 |
| Fig. S12: Extracted ion LC-HRMS chromatograms of nicotine-related compounds in THP-1 cell extract following cation-exchange SPE | S10 |
| Fig. S13: Mirror plots from Compound Discoverer software showing HRMS/MS spectra of methylthioadenosine, L-glutamate and cytosine | S11 |
| Fig. S14: HRMS/MS spectra from data-dependent analysis of uric acid | S12 |
| Fig. S15: Box and whisker plot for cytosine; all data | S13 |
| Fig. S16: Box and whisker plot for uric acid; all data | S14 |
| Fig. S17: Box and whisker plot for methylthioadenosine; all data | S15 |
| Fig. S18: Box and whisker plot for L-glutamate; all data | S16 |
| Table S1a–S1e: List of reference metabolites that were included in an in-house library | S17–S21 |
| Table S2: Complete list of differential metabolites in THP-1 cell extracts (1 h exposure) for which no reference standards were available | S22 |
| Table S3: Complete list of differential metabolites in THP-1 medium extracts (1 h exposure) for which no reference standards were available | S24 |
| Table S4: Complete list of differential metabolites in THP-1 medium extracts (4 h exposure) for which no reference standards were available | S26 |
| Table S5a–S5b: *P*-values from paired *t*-tests of amino acid levels, selected amino acid ratios, and sums of amino acids | S25–S27 |
| Table S6a–S6b: Explanations for the diagnostic relevance of changes in amino acid levels, amino acid and polyamine ratios, and sums of amino acids and polyamines | S27–S28 |

**Table of Contents**


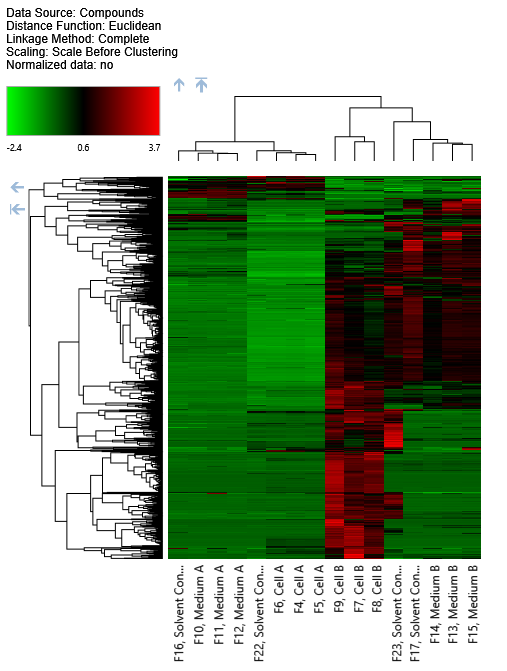


**Figure S1.** Hierarchical clustering analysis of raw data from an initial trial that was aimed at testing the necessity of up-concentration of cell extracts and medium samples. Labels are F16, solvent control for medium sample preparation without up-concentration; F10–F12, medium samples without up-concentration; F22, solvent control for cell extract without up-concentration; F4–F6, cell extracts without up-concentration; F7–F9, up-concentrated cell extracts; F23, solvent control for up-concentrated cell extract; F17, solvent control for up-concentrated medium sample; F13–F15, up-concentrated medium samples.


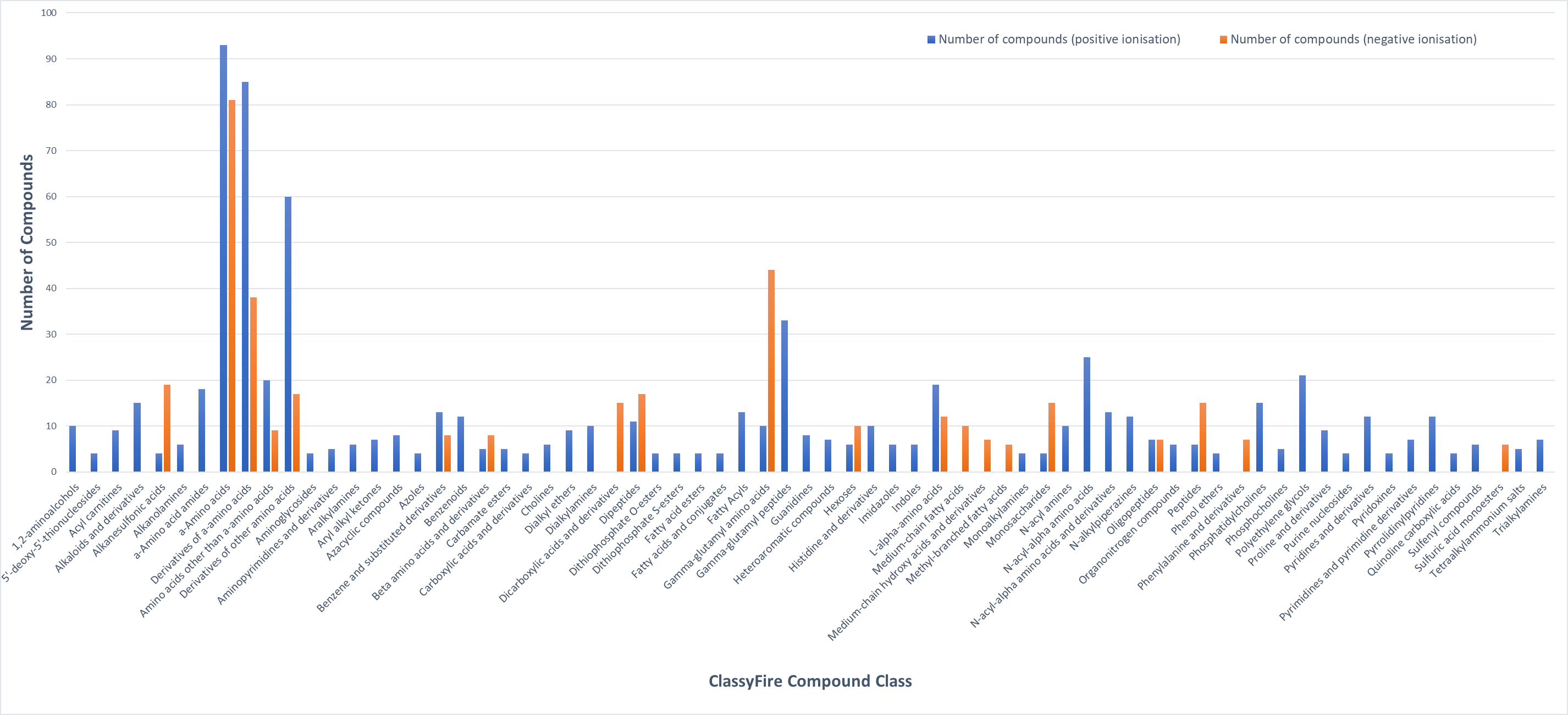


**Figure S2.** Automated chemical class assignment of the 532 compounds that were present in at least 80% of the samples in one group using ClassyFire.


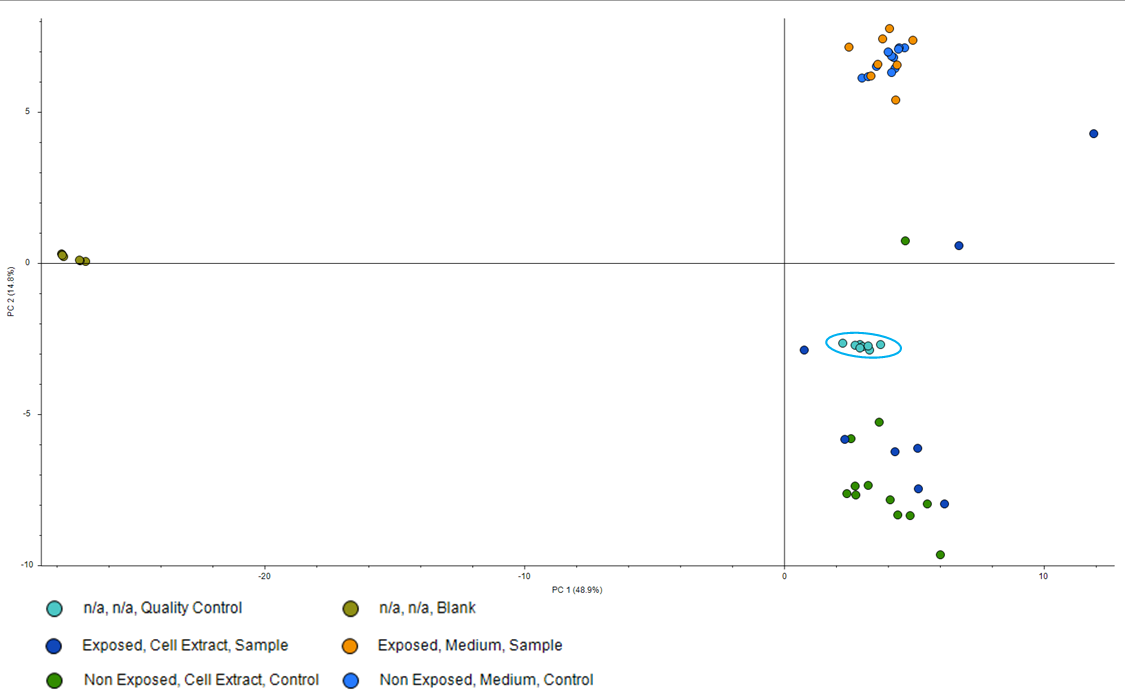


**Figure S3.** Scores plot from principal component analysis of the whole dataset including solvent control and quality control samples. The quality control samples are emphasized with an ellipse, and their overlapping scores show the absence of instrumental drift.


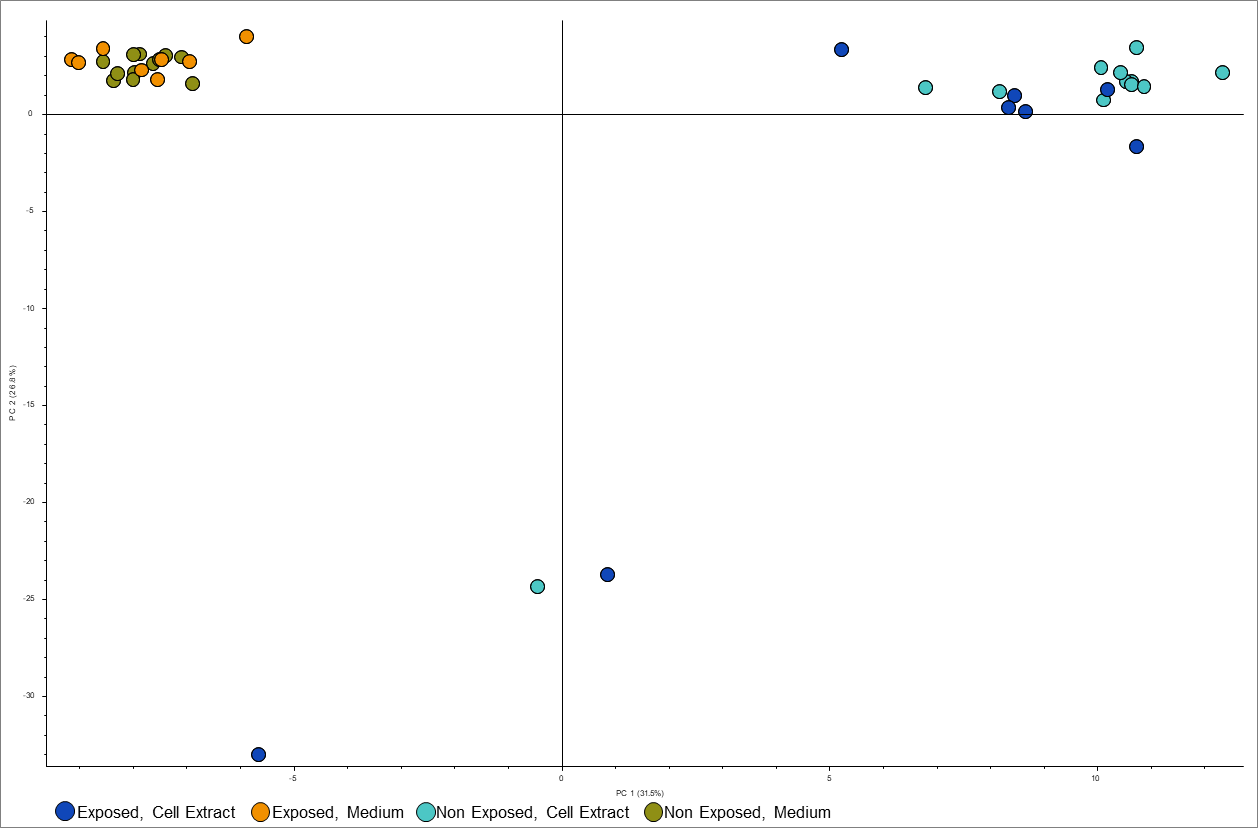


**Figure S4.** Scores plot from principal component analysis of the whole dataset excluding solvent control and quality control samples.


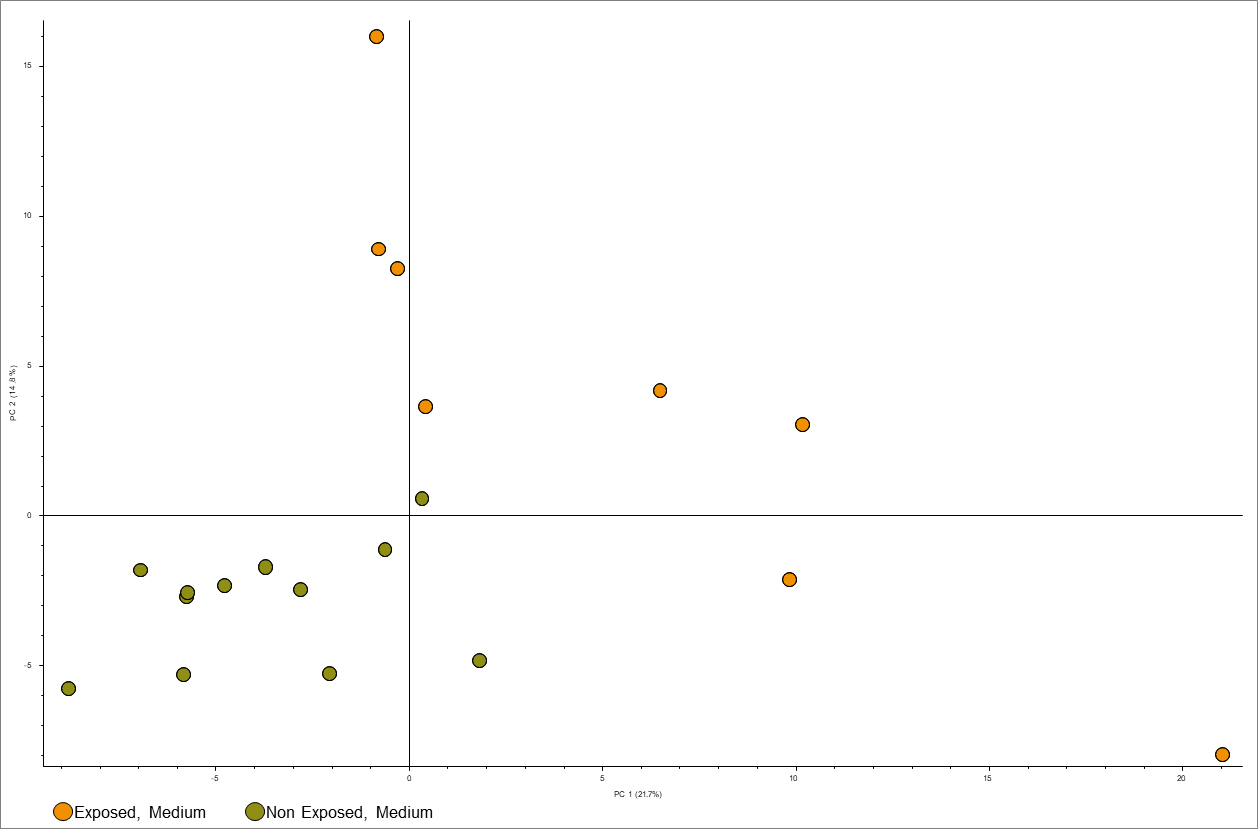


**Figure S5.** Scores plot from principal component analysis of the cell culture medium samples only.


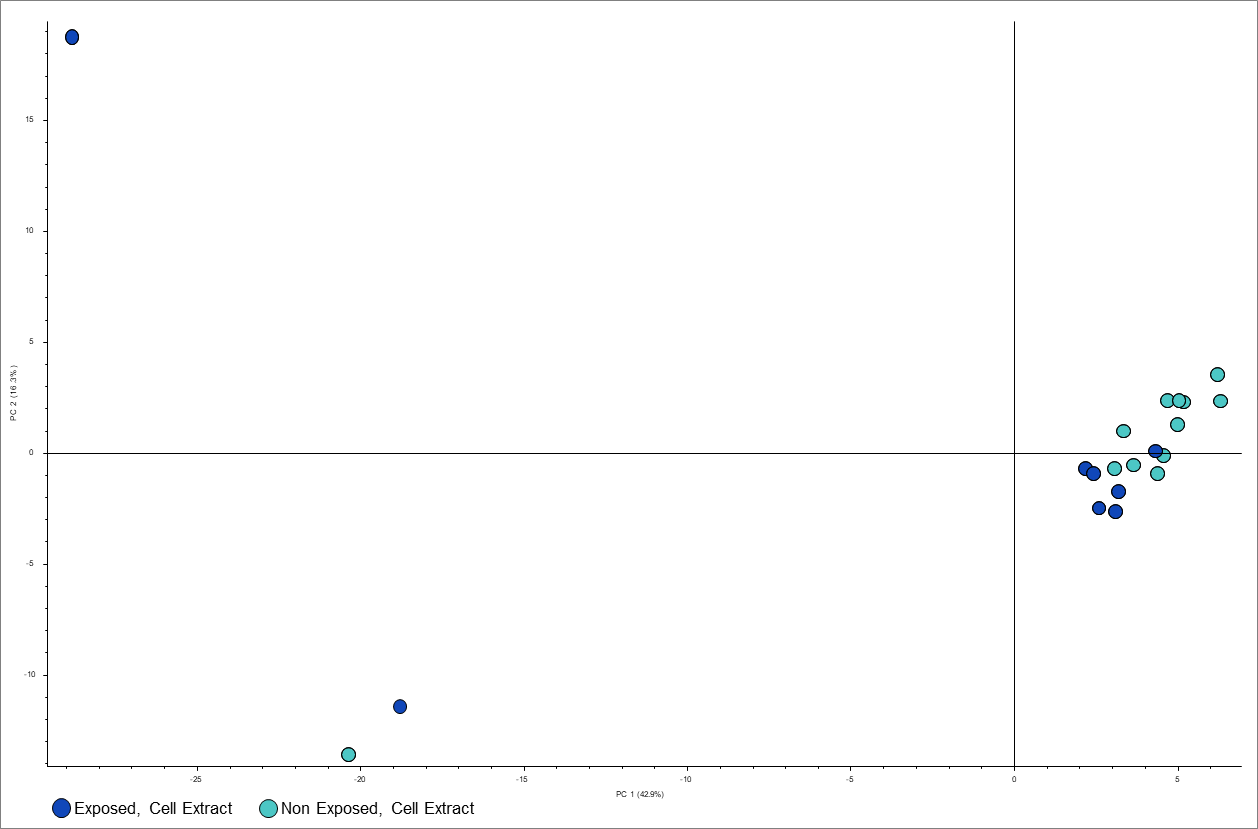


**Figure S6.** Scores plot from principal component analysis of the cell extract samples only.

**
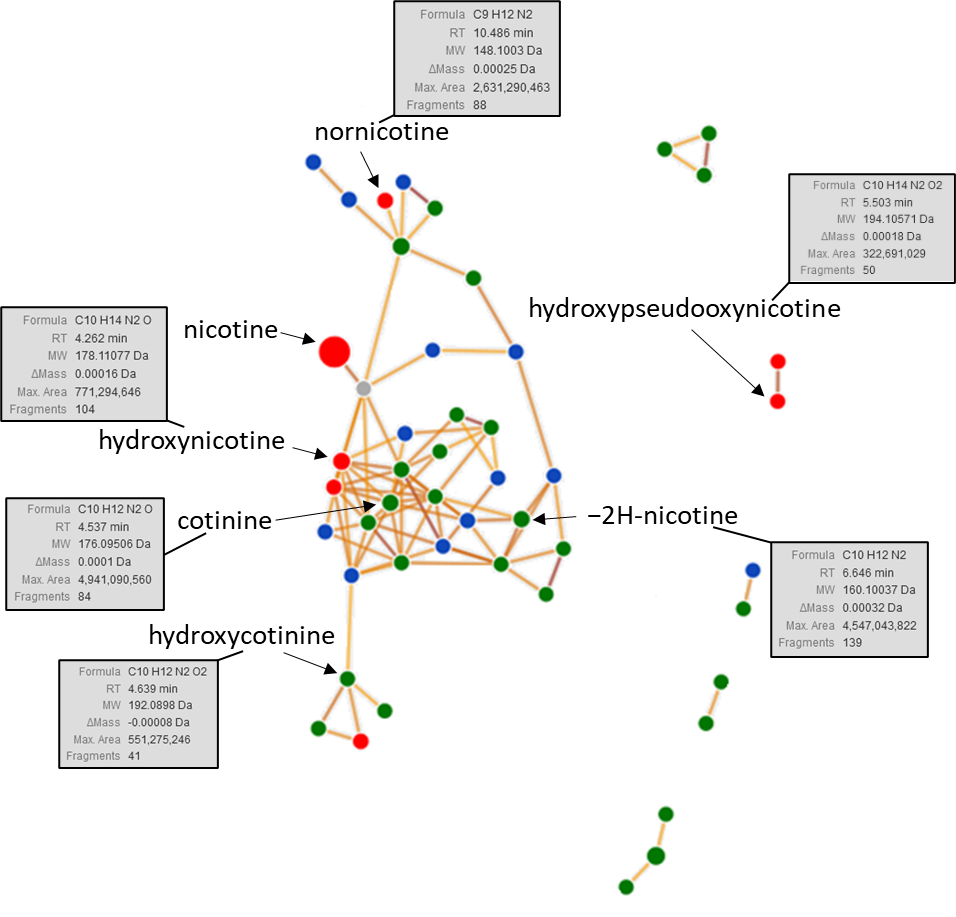
**

**Figure S7.** Example of a nicotine molecular network from Compound Discoverer software based on the spectral similarity of compounds in THP-1 cell extracts and culture medium used to annotate putative nicotine biotransformation products. Threshold settings were: score, 50; coverage, 70 and matched fragments, 30. Only compounds that were identical to reference standards or with MS/MS spectra that supported nicotine-related metabolites are labelled.


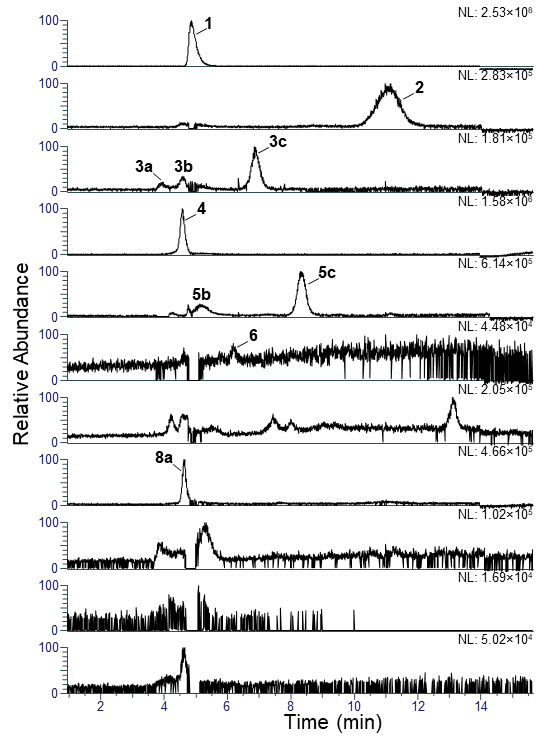


**Figure S8.** Extracted ion LC-HRMS chromatograms (± 5 ppm) of the protonated molecules for nicotine and nicotine metabolites in THP-1 cell extracts prepared by sonication-assisted cell lysis and SPE using Oasis MCX mixed-mode cation exchange cartridges. Individual *m/z* traces correspond to those shown in Figure 2 in the main manuscript.

**Figure S9.** Scores plot from orthogonal partial least squares discriminant analysis (OPLS-DA) of the cell extract samples after an exposure time of 1 h. Labels refer to: CEN, extract from nicotine-exposed cells; CTEN, extract from control cells.

**Figure S10.** Scores plot from orthogonal partial least squares discriminant analysis (OPLS-DA) of the cell extract samples after an exposure time of 4 h. Labels refer to: CEN, extract from nicotine-exposed cells; CTEN, extract from control cells.

**Figure S11.** Scores plot from orthogonal partial least squares discriminant analysis (OPLS-DA) of the medium samples after an exposure time of 1 h. Labels refer to: CMN, medium from nicotine-exposed cells; CTMN, medium from control cells.

**Figure S12.** Scores plot from orthogonal partial least squares discriminant analysis (OPLS-DA) of the medium samples after an exposure time of 4 h. Labels refer to: CMN, medium from nicotine-exposed cells; CTMN, medium from control cells.


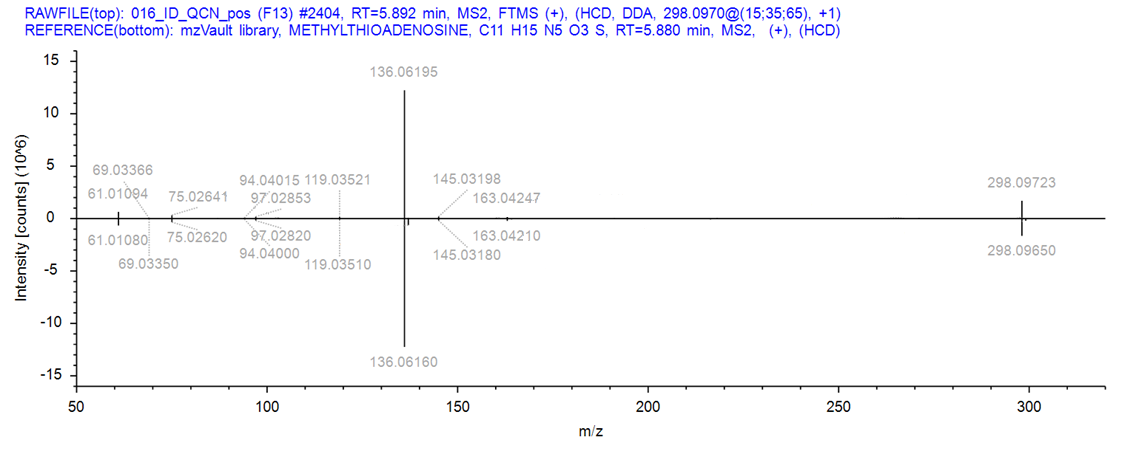


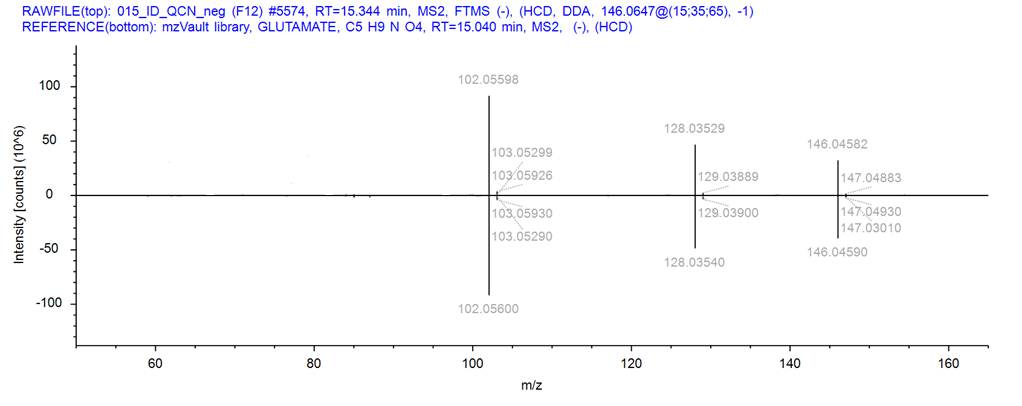


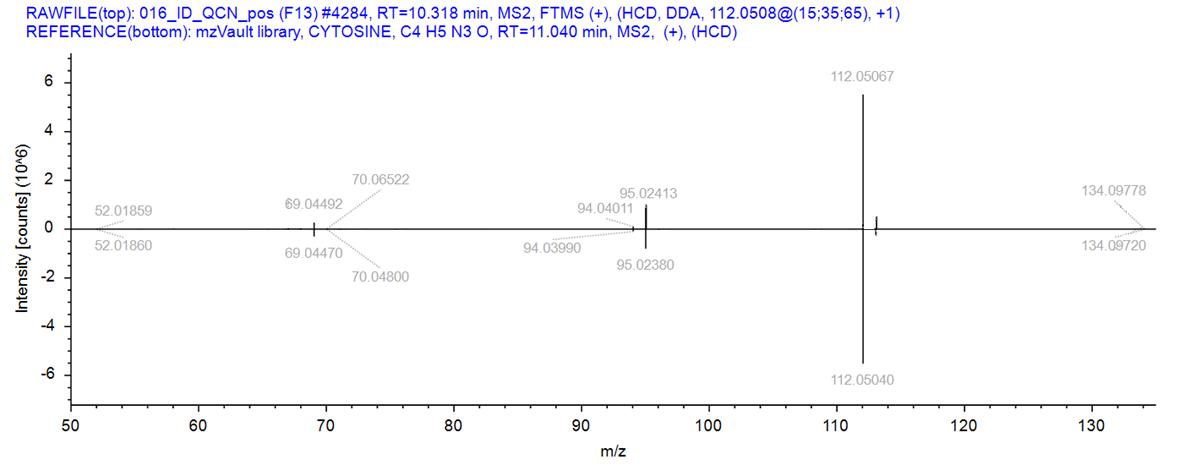


**Figure S13.** Mirror plots from Compound Discoverer software showing HRMS/MS spectra of discriminating and identified compounds (methylthioadenosine, top; L-glutamate, middle; cytosine, bottom) in a pooled THP-1 quality control sample. Reference spectra are shown in the lower trace.


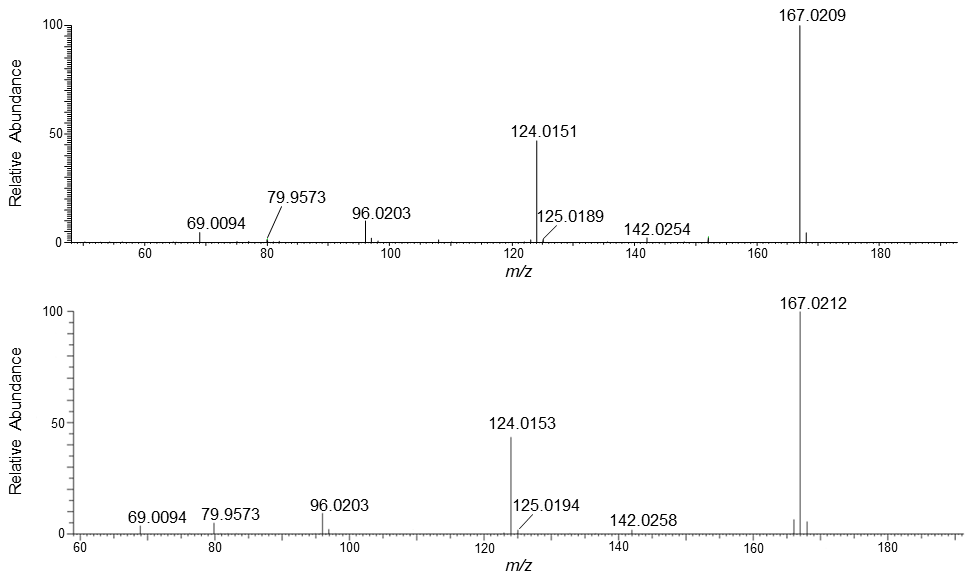


**Figure S14.** HRMS/MS spectra from data-dependent analysis of uric acid ([M−H]^−^) in a pooled THP-1 quality control sample (above) and a reference standard (below).


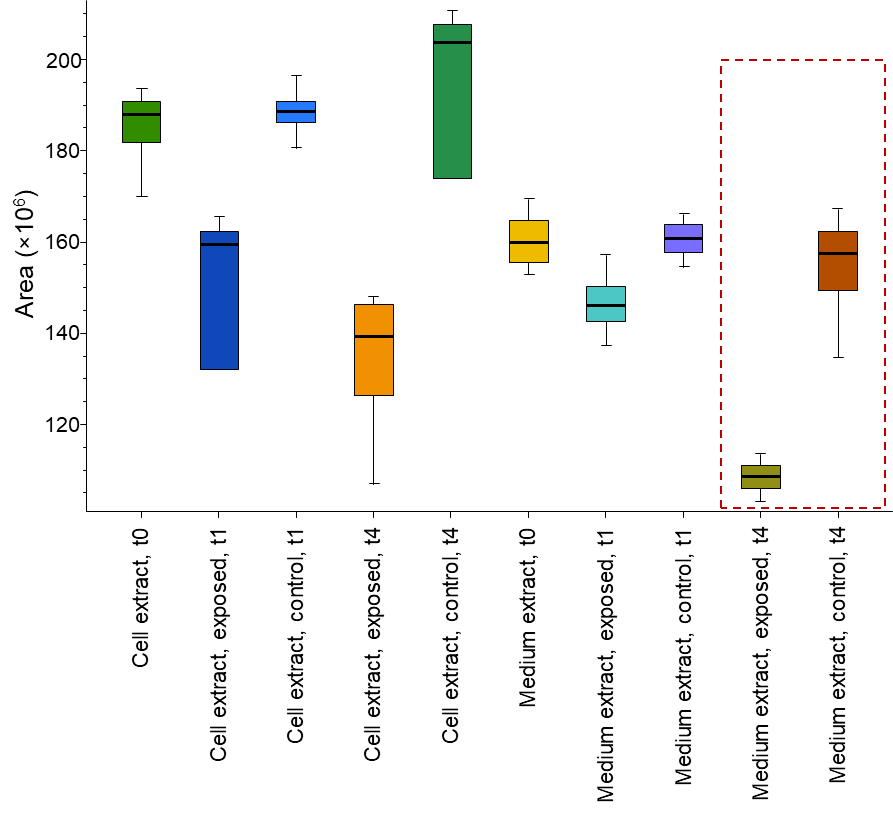


**Figure S15.** Box and whisker plot for peak areas of the protonated molecules of cytosine from the entire data set. The 4-h-exposure (medium extracts) is emphasized as the metabolite differentiated the medium samples from exposed and unexposed cells at this time point both by multivariate and univariate statistics.


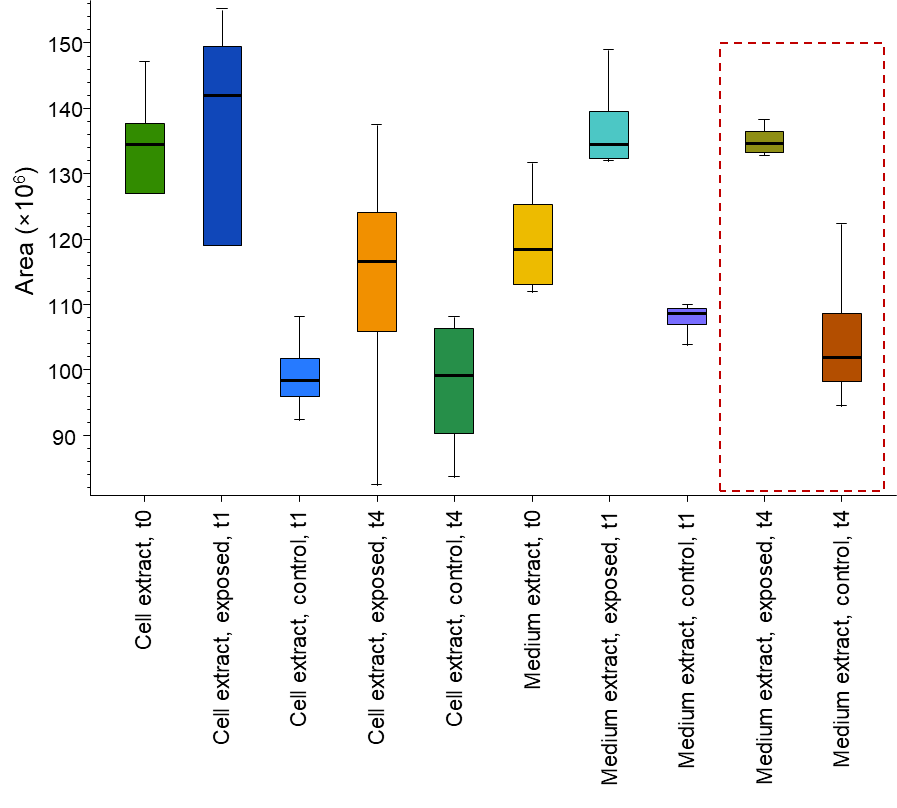


**Figure S16.** Box and whisker plot for peak areas of the deprotonated molecules of uric acid from the entire data set. The 4-h-exposure (medium extracts) is emphasized as the metabolite differentiated the medium samples from exposed and unexposed cells at this time point both by multivariate and univariate statistics.

**
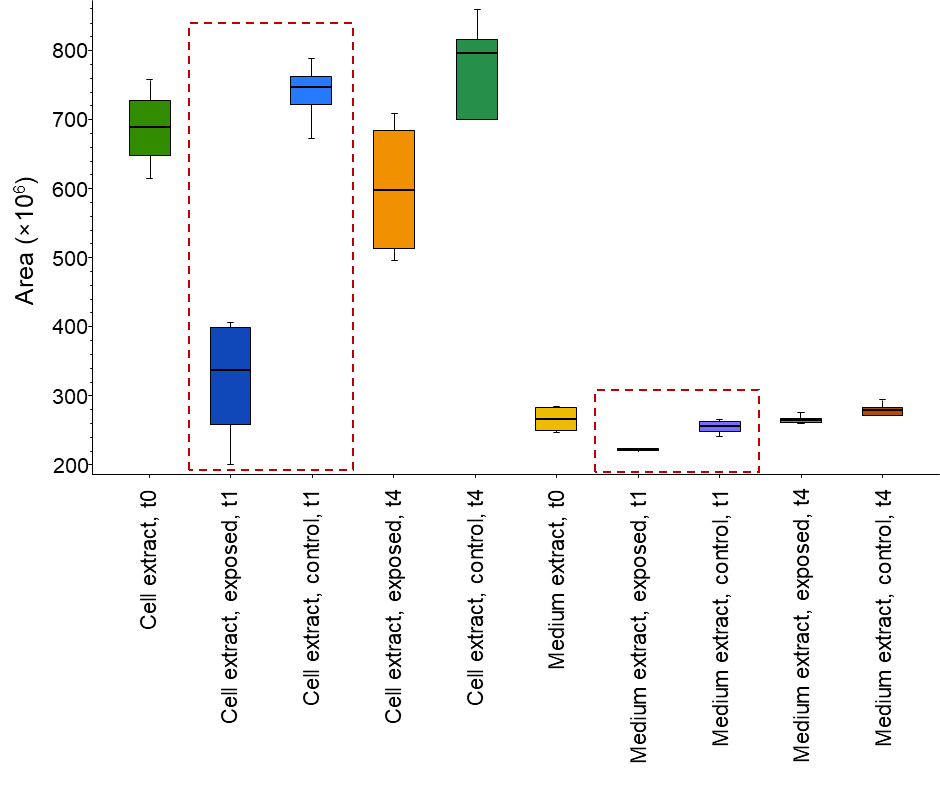
Figure S17.** Box and whisker plot for peak areas of the protonated molecules of methylthioadenosine from the entire data set. The 1-h-exposure is emphasized as the metabolite differentiated exposed and unexposed cells at this time point in cell extracts and medium both by multivariate and univariate statistics.


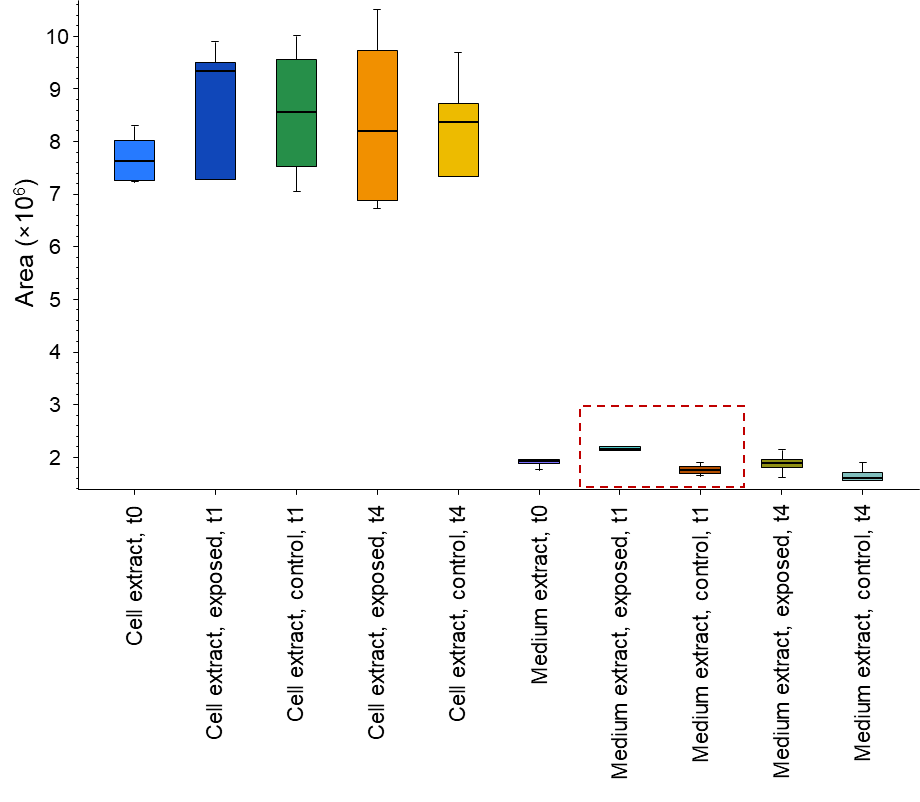


**Figure S18.** Box and whisker plot for peak areas of the protonated molecules of L-glutamate from the entire data set. The 1-h-exposure (medium extracts) is emphasized as the metabolite differentiated the medium samples from exposed and unexposed cells at this time point both by multivariate and univariate statistics.

| **Metabolite name** | **Formula** |  | **Metabolite name** | **Formula** |
| --- | --- | --- | --- | --- |
| BETA-NICOTINAMIDE ADENINE DINUCLEOTIDE | C21H27N7O14P2 |  | URACIL | C4H4N2O2 |
| TRANS-ACONITATE | C6H6O6 |  | ASPARTATE | C4H7NO4 |
| URATE | C5H4N4O3 |  | DEOXYCYTIDINE MONOPHOSPHATE | C9H14N3O7P |
| GLUTAMINE | C5H10N2O3 |  | HYPOXANTHINE | C5H4N4O |
| HYPOTAURINE | C2H7NO2S |  | CREATINE | C4H9N3O2 |
| N-METYL-ALANINE | C4H9NO2 |  | L-DOPA | C9H11NO4 |
| CITRATE | C6H8O7 |  | ISOCITRATE | C6H8O7 |
| THREONINE | C4H9NO3 |  | FOLATE | C19H19N7O6 |
| PURINE | C5H4N4 |  | NICOTINAMIDE MONONUCLEOTIDE | C11H15N2O8P |
| N-ACETYLNEURAMINATE | C11H19NO9 |  | 3-METHYL-L-HISTIDINE | C7H11N3O2 |
| N-ACETYLMANNOSAMINE | C8H15NO6 |  | TYROSINE | C9H11NO3 |
| CYTIDINE | C9H13N3O5 |  | GLYCEROL | C3H8O3 |
| CYTOSINE | C4H5N3O |  | ASPARAGINE | C4H8N2O3 |
| ISOLEUCINE | C6H13NO2 |  | VALINE | C5H11NO2 |
| SERINE | C3H7NO3 |  | GUANINE | C5H5N5O |
| CITRULLINE | C6H13N3O3 |  | HOMOSERINE | C4H9NO3 |
| TAURINE | C2H7NO3S |  | PYRIDOXINE | C8H11NO3 |
| N-ACETYLTRYPTOPHAN | C13H14N2O3 |  | DEOXYADENOSINE MONOPHOSPHATE | C10H14N5O6P |
| NICOTINATE | C6H5NO2 |  | DIAMINOPIMELATE | C7H14N2O4 |
| INOSINE | C10H12N4O5 |  | THEOPHYLLINE | C7H8N4O2 |
| GAMMA-AMINOBUTYRATE | C4H9NO2 |  | LEUCINE | C6H13NO2 |
| GLUTAMATE | C5H9NO4 |  | TREHALOSE | C12H22O11 |
| MALONATE | C3H4O4 |  | AMINOADIPATE | C6H11NO4 |
| GUANIDINOACETATE | C3H7N3O2 |  | DEOXYCYTIDINE | C9H13N3O4 |
| P-HYDROXYPHENYLACETATE | C8H8O3 |  | NORADRENALINE | C8H11NO3 |
| N-ACETYLGLUCOSAMINE | C8H15NO6 |  | GLUCOSAMINE 6-PHOSPHATE | C6H14NO8P |
| GLYCOLATE | C2H4O3 |  | TARTRATE | C4H6O6 |
| SARCOSINE | C3H7NO2 |  | 3-DEHYDROSHIKIMATE | C7H8O5 |
| CREATININE | C4H7N3O |  | CAFFEINE | C8H10N4O2 |
| QUINATE | C7H12O6 |  | HOMOCYSTEINE | C4H9NO2S |
| DIHYDROOROTATE | C5H6N2O4 |  | BETAINE | C5H11NO2 |
| GLYCINE | C2H5NO2 |  | THREITOL | C4H10O4 |
| XANTHINE | C5H4N4O2 |  | GLUCOSAMINE | C6H13NO5 |
| CYSTINE | C6H12N2O4S2 |  | TRYPTOPHAN | C11H12N2O2 |
| METHIONINE | C5H11NO2S |  | 3-SULFINOALANINE | C3H7NO4S |
| 2-PHOSPHOGLYCERATE | C3H7O7P |  | O-SUCCINYL-HOMOSERINE | C8H13NO6 |
| METHYLTHIOADENOSINE | C11H15N5O3S |  | ALLANTOIN | C4H6N4O3 |
| THYMIDINE | C10H14N2O5 |  | GLYCERALDEHYDE | C3H6O3 |
| CYS-GLY | C5H10N2O3S |  | D-GLUCURONOLACTONE | C6H8O6 |
| AMINOISOBUTANOATE | C4H9NO2 |  | (2-AMINOETHYL)PHOSPHONATE | C2H8NO3P |
| GULOSE | C6H12O6 |  | 2,5-DIHYDROBENZOIC ACID | C7H6O4 |
| CARNOSINE | C9H14N4O3 |  | PARAXANTHINE | C7H8N4O2 |
| SHIKIMATE | C7H10O5 |  | DEOXYGUANOSINE-MONOPHOSPHATE | C10H14N5O7P |
| DIETHANOLAMINE | C4H11NO2 |  | GLUCURONATE | C6H10O7 |
| URIDINE MONOPHOSPHATE | C9H13N2O9P |  | 2-DEOXY-D-GLUCOSE | C6H12O5 |
| PROLINE | C5H9NO2 |  | 1-METHYL-L-HISTIDINE | C7H11N3O2 |
| THYMINE | C5H6N2O2 |  | GALACTITOL | C6H14O6 |
| SUCCINATE SEMIALDEHYDE | C4H6O3 |  | OXOPROLINE | C5H7NO3 |
| URIDINE | C9H12N2O6 |  | 4-PYRIDOXATE | C8H9NO4 |
| SUCCINATE | C4H6O4 |  | QUINOLINATE | C7H5NO4 |
| GUANOSINE | C10H13N5O5 |  | 1-METHYLADENOSINE | C11H15N5O4 |
| DIHYDROURACIL | C4H6N2O2 |  | DOPAMINE | C8H11NO2 |
| MALATE | C4H6O5 |  | ADENOSINE-MONOPHOSPHATE | C10H14N5O7P |
| PHENYLALANINE | C9H11NO2 |  | LYSINE | C6H14N2O2 |

**Table S1a.** List of reference metabolites that were included in an in-house library.

| **Metabolite name** | **Formula** |  | **Metabolite name** | **Formula** |
| --- | --- | --- | --- | --- |
| DEOXYURIDINE | C9H12N2O5 |  | PHOSPHOENOLPYRUVATE | C3H5O6P |
| GLUCONATE | C6H12O7 |  | MANNOSE 6-PHOSPHATE | C6H13O9P |
| UROCANATE | C6H6N2O2 |  | 3-PHOSPHOGLYCERATE | C3H7O7P |
| KYNURENINE | C10H12N2O3 |  | GLYCERATE | C3H6O4 |
| PYROGLUTAMATE | C5H7NO3 |  | CYTIDINE 2',3'-CYCLIC PHOSPHATE | C9H12N3O7P |
| 4-ACETAMIDOBUTANOATE | C6H11NO3 |  | N,N,N-TRIMETHYLLYSINE | C9H20N2O2 |
| TRANS-1,2-CYCLOHEXANEDIOL | C6H12O2 |  | RIBOFLAVIN | C17H20N4O6 |
| CITICOLINE | C14H27N4O11P2 |  | URIDINE DIPHOSPHATE GLUCOSE | C15H24N2O17P2 |
| ARGININE | C6H14N4O2 |  | METHYL GALACTOSIDE | C7H14O6 |
| PHOSPHOSERINE | C3H8NO6P |  | PYRIDOXAL-PHOSPHATE | C8H10NO6P |
| 1-AMINOCYCLOPROPANECARBOXYLATE | C4H7NO2 |  | DIHYDROXYACETONE PHOSPHATE | C3H7O6P |
| GLUTARYLCARNITINE | C12H21NO6 |  | L-CARNITINE | C7H15NO3 |
| CYSTATHIONINE | C7H14N2O4S |  | P-OCTOPAMINE | C8H11NO2 |
| NORVALINE | C5H11NO2 |  | N-ALPHA-ACETYLLYSINE | C8H16N2O3 |
| 3-HYDROXYMETHYLGLUTARATE | C6H10O5 |  | URIDINE DIPHOSPHATEGALACTOSE | C15H24N2O17P2 |
| PHOSPHONOACETATE | C2H5O5P |  | O-PHOSPHOETHANOLAMINE | C2H8NO4P |
| TRANS-4-HYDROXY-L-PROLINE | C5H9NO3 |  | CYTIDINE MONOPHOSPHATE | C9H14N3O8P |
| N-METHYLASPARTATE | C5H9NO4 |  | GUANOSINE DIPHOSPHATE MANNOSE | C16H25N5O16P2 |
| GALACTARATE | C6H10O8 |  | ADP-GLUCOSE | C16H25N5O15P2 |
| ALPHA-HYDROXYISOBUTYRATE | C4H8O3 |  | FRUCTOSE 6-PHOSPHATE | C6H13O9P |
| FUCOSE | C6H12O5 |  | ADENOSINE 3',5'-DIPHOSPHATE | C10H15N5O10P2 |
| HOMOCYSTINE | C8H16N2O4S2 |  | 3-NITRO-L-TYROSINE | C9H10N2O5 |
| N-METHYLGLUTAMATE | C6H11NO4 |  | OROTATE | C5H4N2O4 |
| D-ORNITHINE | C5H12N2O2 |  | LAUROYLCARNITINE | C19H38NO4 |
| XANTHOSINE | C10H12N4O6 |  | 1-METHYLNICOTINAMIDE | C7H9N2O |
| THYROTROPIN RELEASING HORMONE | C16H22N6O4 |  | PYRIDOXAMINE | C8H12N2O2 |
| CYSTEATE | C3H7NO5S |  | 5-AMINOLEVULINATE | C5H9NO3 |
| GLYCOLALDEHYDE DIMER | C4H8O4 |  | DEOXYURIDINE-MONOPHOSPHATE | C9H13N2O8P |
| N-ACETYLASPARAGINE | C6H10N2O4 |  | 5'-DEOXYADENOSINE | C10H13N5O3 |
| PIPECOLATE | C6H11NO2 |  | XANTHOSINE-MONOPHOSPHATE | C10H13N4O9P |
| GLUCOSE 6-PHOSPHATE | C6H13O9P |  | DEOXYGUANOSINE | C10H13N5O4 |
| NADP | C21H28N7O17P3 |  | O-PHOSPHOSERINE | C3H8NO6P |
| 6-PHOSPHOGLUCONATE | C6H13O10P |  | S-ADENOSYLHOMOCYSTEINE | C14H20N6O5S |
| ISOPENTENYL PYROPHOSPHATE | C5H12O7P2 |  | N-ACETYLMETHIONINE | C7H13NO3S |
| DTDP-D-GLUCOSE | C16H26N2O16P2 |  | CARBAMOYL PHOSPHATE | CH4NO5P |
| N-ACETYLGLYCINE | C4H7NO3 |  | AICAR | C9H15N4O8P |
| 2-KETO-3-DEOXY-D-GLUCONIC ACID | C6H10O6 |  | URIDINE DIPHOSPHATE-N-ACETYLGALACTOSAMINE | C17H27N3O17P2 |
| D-SEDOHEPTULOSE | C7H14O7 |  | CYCLIC GMP | C10H12N5O7P |
| N-ACETYLASPARTATE | C6H9NO5 |  | HOMOCYSTEINE THIOLACTONE | C4H7NOS |
| PALMITOYLCARNITINE | C23H46NO4 |  | ADENINE | C5H5N5 |
| NORSPERMIDINE | C6H17N3 |  | HISTAMINE | C5H9N3 |
| NICOTINAMIDE HYPOXANTHINE DINUCLEOTIDE | C21H26N6O15P2 |  | INDOXYL SULFATE | C8H7NO4S |
| S-ADENOSYLMETHIONINE | C15H23N6O5S |  | ETHYL 3-UREIDOPROPIONATE | C6H12N2O3 |
| ERYTHRITOL | C4H10O4 |  | NORMETANEPHRINE | C9H13NO3 |
| GLUCOSAMINATE | C6H13NO6 |  | URIDINE DIPHOSPHATE-N-ACETYLGLUCOSAMINE | C17H27N3O17P2 |
| PUTRESCINE | C4H12N2 |  | GLUTATHIONE REDUCED | C10H17N3O6S |
| THIAMINE | C12H17N4OS |  | N,N-DIMETHYLARGININE | C8H18N4O2 |
| DEOXYCARNITINE | C7H15NO2 |  | CYTIDINE DIPHOSPHATE | C9H15N3O11P2 |
| ADENOSINE 2',3'-CYCLIC PHOSPHATE | C10H12N5O6P |  | DEOXYRIBOSE | C5H10O4 |
| MEVALOLACTONE | C6H10O3 |  | HYDROXYPYRUVATE | C3H4O4 |
| GALACTOSE 1-PHOSPHATE | C6H13O9P |  | D-MANNOSAMINE | C6H13NO5 |
| PHOSPHORYLCHOLINE | C5H14NO4P |  | TRIMETHYLACETIC ACID | C5H10O2 |
| O-ACETYLCARNITINE | C9H17NO4 |  | URACIL 5-CARBOXYLATE | C5H4N2O4 |
| 5-METHYLCYTOSINE | C5H7N3O |  | GLYOXYLATE | C2H2O3 |

**Table S1b.** List of reference metabolites that were included in an in-house library, continued.

| **Metabolite name** | **Formula** |  | **Metabolite name** | **Formula** |
| --- | --- | --- | --- | --- |
| GUANOSINE MONOPHOSPHATE | C10H14N5O8P |  | CDP-ETHANOLAMINE | C11H20N4O11P2 |
| N-ACETYLALANINE | C5H9NO3 |  | MESOXALATE | C3H2O5 |
| 4-GUANIDINOBUTANOATE | C5H11N3O2 |  | TRIGONELLINE | C7H7NO2 |
| DEOXYADENOSINE | C10H13N5O3 |  | EPINEPHRINE | C9H13NO3 |
| 2-AMINOISOBUTYRATE | C4H9NO2 |  | 3,4-DIHYDROXYPHENYLGLYCOL | C8H10O4 |
| ANILINE-2-SULFONATE | C6H7NO3S |  | CADAVERINE | C5H14N2 |
| S-CARBOXYMETHYLCYSTEINE | C5H9NO4S |  | 2-HYDROXYBUTYRATE | C4H8O3 |
| N-ACETYLPUTRESCINE | C6H14N2O |  | STACHYOSE | C24H42O21 |
| N-ACETYLGALACTOSAMINE | C8H15NO6 |  | 4-HYDROXY-L-PHENYLGLYCINE | C8H9NO3 |
| N-ACETYLGLUTAMATE | C7H11NO5 |  | N-ACETYLSERINE | C5H9NO4 |
| 2,4-DIHYDROXYPTERIDINE | C6H4N4O2 |  | DEOXYCYTIDINE-DIPHOSPHATE | C9H15N3O10P2 |
| 6-HYDROXYNICOTINATE | C6H5NO3 |  | 2,3-BUTANEDIOL | C4H10O2 |
| N-ACETYLCYSTEINE | C5H9NO3S |  | D-RIBOSE 5-PHOSPHATE | C5H11O8P |
| INOSINE-MONOPHOSPHATE | C10H13N4O8P |  | HYDROXYKYNURENINE | C10H12N2O4 |
| PANTOTHENATE | C9H17NO5 |  | GALACTOSAMINE | C6H13NO5 |
| RHAMNOSE | C6H12O5 |  | GLYCEROL 3-PHOSPHATE | C3H9O6P |
| RAFFINOSE | C18H32O16 |  | CYANOCOBALAMIN | C63H88CoN14O14P |
| 2-ACETAMIDO-2-DEOXY-BETA-D-GLUCOSYLAMINE | C8H16N2O5 |  | METHYGLUTARATE | C6H10O4 |
| THIAMINE PYROPHOSPHATE | C12H18N4O7P2S |  | XANTHURENATE | C10H7NO4 |
| HISTIDINOL | C6H11N3O |  | INDOXYL Î²-GLUCOSIDE | C14H17NO6 |
| THYMIDINE-MONOPHOSPHATE | C10H15N2O8P |  | SORBATE | C6H8O2 |
| UREIDOPROPIONATE | C4H8N2O3 |  | MONOETHYLMALONATE | C5H8O4 |
| 5-AMINOPENTANOATE | C5H11NO2 |  | GLUCONOLACTONE | C6H10O6 |
| NORLEUCINE | C6H13NO2 |  | 4-HYDROXYBENZOATE | C7H6O3 |
| N-FORMYLGLYCINE | C3H5NO3 |  | TYRAMINE | C8H11NO |
| ADENOSINE | C10H13N5O4 |  | CORTISOL | C21H30O5 |
| SACCHARATE | C6H10O8 |  | PRENOL | C5H10O |
| N1-ACETYLSPERMINE | C12H28N4O |  | TRIMETHYLAMINE | C3H9N |
| 3-METHOXYTYROSINE | C10H13NO4 |  | ISOBUTYRATE | C4H8O2 |
| LACTOSE | C12H22O11 |  | MELATONIN | C13H16N2O2 |
| 3-HYDROXYBUTANOATE | C4H8O3 |  | MALEATE | C4H4O4 |
| 4-IMIDAZOLEACETATE | C5H6N2O2 |  | PENTANOATE | C5H10O2 |
| GALACTURONATE | C6H10O7 |  | BILIRUBIN | C33H36N4O6 |
| CYCLIC AMP | C10H12N5O6P |  | NICOTINE | C10H14N2 |
| GLUCOSAMINE 6-SULFATE | C6H13NO8S |  | PREGNENOLONE SULFATE | C21H32O5S |
| 5-HYDROXYTRYPTOPHAN | C11H12N2O3 |  | KYNURENATE | C10H7NO3 |
| THEOBROMINE | C7H8N4O2 |  | ACETOIN | C4H8O2 |
| 3-METHYLHISTAMINE | C6H11N3 |  | BETA-ALANINE | C3H7NO2 |
| CHOLINE | C5H14NO |  | N-ACETYLPHENYLALANINE | C11H13NO3 |
| METHYL 4-AMINOBUTYRATE | C5H11NO2 |  | N-ACETYLPROLINE | C7H11NO3 |
| N-FORMYL-L-METHIONINE | C6H11NO3S |  | 3,5-DIIODO-L-TYROSINE | C9H9I2NO3 |
| ACETYLCHOLINE | C7H16NO2 |  | MANDELATE | C8H8O3 |
| OXALATE | C2H2O4 |  | TRYPTAMINE | C10H12N2 |
| GUANIDINOSUCCINATE | C5H9N3O4 |  | 4-AMINOBENZOATE | C7H7NO2 |
| BILIVERDIN | C33H34N4O6 |  | GLUTARATE | C5H8O4 |
| 5-HYDROXYLYSINE | C6H14N2O3 |  | 5-VALEROLACTONE | C5H8O2 |
| HISTIDINE | C6H9N3O2 |  | CAFFEATE | C9H8O4 |
| ALLOTHREONINE | C4H9NO3 |  | LUMICHROME | C12H10N4O2 |
| PHOSPHOCREATINE | C4H10N3O5P |  | L-TRYPTOPHANAMIDE | C11H13N3O |
| SPERMIDINE | C7H19N3 |  | PHENYLETHANOLAMINE | C8H11NO |
| ADENOSINE DIPHOSPHATE RIBOSE | C15H23N5O14P2 |  | THIOPURINE S-METHYLETHER | C6H6N4S |
| CITRAMALATE | C5H8O5 |  | 2-HYDROXY-4-(METHYLTHIO)BUTANOATE | C5H10O3S |
| ANSERINE | C10H16N4O3 |  | N-METHYLTRYPTAMINE | C11H14N2 |
| OPHTHALMATE | C11H19N3O6 |  | 2,3-DIHYDROXYBENZOATE | C7H6O4 |

**Table S1c.** List of reference metabolites that were included in an in-house library, continued.

| **Metabolite name** | **Formula** |  | **Metabolite name** | **Formula** |
| --- | --- | --- | --- | --- |
| INDOLE-3-ETHANOL | C10H11NO |  | N-ACETYLSEROTONIN | C12H14N2O2 |
| FERULATE | C10H10O4 |  | N-ETHYL-5-METHYL-2-(1-METHYLETHYL)-CYCLOHEXANECARBOXAMIDE | C13H25NO |
| GLYCOCHOLATE | C26H43NO6 |  | ITACONATE | C5H6O4 |
| GLYCOCHENODEOXYCHOLATE | C26H43NO5 |  | PHENYLACETALDEHYDE | C8H8O |
| 10-HYDROXYDECANOATE | C10H20O3 |  | SUBERATE | C8H14O4 |
| DIDECANOYL-GLYCEROPHOSPHOCHOLINE | C28H56NO8P |  | ADIPATE | C6H10O4 |
| 2-HYDROXYPYRIDINE | C5H5NO |  | 3-METHYL-2-OXOVALERATE | C6H10O3 |
| BENZOATE | C7H6O2 |  | PORPHOBILINOGEN | C10H14N2O4 |
| 3-AMINO-5-HYDROXYBENZOATE | C7H7NO3 |  | DIACETYL | C4H6O2 |
| PYROCATECHOL | C6H6O2 |  | PYRUVATE | C3H4O3 |
| 3,4-DIHYDROXYBENZOATE | C7H6O4 |  | TRANS-CINNAMALDEHYDE | C9H8O |
| CYCLOPENTANONE | C5H8O |  | 2,6-DIHYDROXYPYRIDINE | C5H5NO2 |
| PANTOLACTONE | C6H10O3 |  | VANILLIN | C8H8O3 |
| GUAIACOL | C7H8O2 |  | N-ACETYLLEUCINE | C8H15NO3 |
| 2-HYDROXYPHENYLACETATE | C8H8O3 |  | 1-PHENYLETHANOL | C8H10O |
| 3,4-DIHYDROXYPHENYLACETATE | C8H8O4 |  | SALSOLINOL | C10H13NO2 |
| 5-METHYLCYTIDINE | C10H15N3O5 |  | SALICYLAMIDE | C7H7NO2 |
| CORTISOL 21-ACETATE | C23H32O6 |  | 2',4'-DIHYDROXYACETOPHENONE | C8H8O3 |
| N6-(DELTA2-ISOPENTENYL)-ADENINE | C10H13N5 |  | BENZYL ALCOHOL | C7H8O |
| METHYL VANILLATE | C9H10O4 |  | MONOMETHYLGLUTARATE | C6H10O4 |
| LIPOAMIDE | C8H15NOS2 |  | INDOLE-3-METHYL ACETATE | C11H11NO2 |
| 3-HYDROXYANTHRANILATE | C7H7NO3 |  | MEVALONATE | C6H12O4 |
| 3-(4-HYDROXYPHENYL)PYRUVATE | C9H8O4 |  | 3-METHOXY-4-HYDROXYMANDELATE | C9H10O5 |
| HEXANOATE | C6H12O2 |  | HOMOVANILLATE | C9H10O4 |
| METHYLMALONATE | C4H6O4 |  | OXOGLUTARATE | C5H6O5 |
| HIPPURATE | C9H9NO3 |  | PYRIDOXAL | C8H9NO3 |
| 2-QUINOLINECARBOXYLATE | C10H7NO2 |  | SALICYLATE | C7H6O3 |
| SEROTONIN | C10H12N2O |  | SEBACATE | C10H18O4 |
| PTERIN | C6H5N5O |  | ETHYL 3-INDOLEACETATE | C12H13NO2 |
| ETHYLMALONATE | C5H8O4 |  | 3-ALPHA,11-BETA,17,21-TETRAHYDROXY- 5-BETA-PREGNAN-20-ONE | C21H34O5 |
| 3,5-DIIODO-L-THYRONINE | C15H13I2NO4 |  | N,N-DIMETHYL-1,4-PHENYLENEDIAMINE | C8H12N2 |
| FUMARATE | C4H4O4 |  | HOMOGENTISATE | C8H8O4 |
| BENZALDEHYDE | C7H6O |  | INDOLEACETALDEHYDE | C10H9NO |
| 4-HYDROXYBENZALDEHYDE | C7H6O2 |  | 4-HYDROXY-3-METHOXYPHENYLGLYCOL | C9H12O4 |
| 3-(2-HYDROXYPHENYL)PROPANOATE | C9H10O3 |  | 3-HYDROXYPHENYLACETATE | C8H8O3 |
| 3-METHOXYTYRAMINE | C9H13NO2 |  | 4-METHYLCATECHOL | C7H8O2 |
| BENZYLAMINE | C7H9N |  | 3-METHYL-2-OXINDOLE | C9H9NO |
| BETA-GLYCEROPHOSPHATE | C3H9O6P |  | RESORCINOL MONOACETATE | C8H8O3 |
| GLUCOSE 1-PHOSPHATE | C6H13O9P |  | ACETOACETATE | C4H6O3 |
| 2-AMINOPHENOL | C6H7NO |  | 3-METHYLADENINE | C6H7N5 |
| 6-CARBOXYHEXANOATE | C7H12O4 |  | HYDROXYPHENYLLACTATE | C9H10O4 |
| DEHYDROASCORBATE | C6H6O6 |  | BIOTIN | C10H16N2O3S |
| 3-AMINO-4-HYDROXYBENZOATE | C7H7NO3 |  | PYRUVIC ALDEHYDE | C3H4O2 |
| 3,4 DIHYDROXYMANDELATE | C8H8O5 |  | PYRROLE-2-CARBOXYLATE | C5H5NO2 |
| DIHYDROBIOPTERIN | C9H13N5O3 |  | 5-HYDROXYINDOLEACETATE | C10H9NO3 |
| 2-METHOXYESTRONE | C19H24O3 |  | 3-METHYLGLUTACONATE | C6H8O4 |
| AZELATE | C9H16O4 |  | SORBOSE | C6H12O6 |
| OXOADIPATE | C6H8O5 |  | ALLOSE | C6H12O6 |
| 2-METHYLGLUTARATE | C6H10O4 |  | MANNITOL | C6H14O6 |
| 4-QUINOLINECARBOXYLATE | C10H7NO2 |  | MELIBIOSE | C12H22O11 |
| HYDROQUINONE | C6H6O2 |  | XYLITOL | C5H12O5 |
| DETHIOBIOTIN | C10H18N2O3 |  | RIBITOL | C5H12O5 |
| 3-HYDROXYBENZOATE | C7H6O3 |  | MYOINOSITOL | C6H12O6 |
| 2-METHYLBUTANAL | C5H10O |  | MANNOSE | C6H12O6 |

**Table S1d.** List of reference metabolites that were included in an in-house library, continued.

| **Metabolite name** | **Formula** |  | **Metabolite name** | **Formula** |
| --- | --- | --- | --- | --- |
| XYLOSE | C5H10O5 |  | 1-HYDROXY-2-NAPHTHOATE | C11H8O3 |
| SUCROSE | C12H22O11 |  | PHENYLPYRUVATE | C9H8O3 |
| GALACTOSE | C6H12O6 |  | CHOLATE | C24H40O5 |
| ALPHA-D-GLUCOSE | C6H12O6 |  | QUINOLINE | C9H7N |
| SORBITOL | C6H14O6 |  | DOCOSAHEXAENOATE | C22H32O2 |
| RIBOSE | C5H10O5 |  | DIETHYL 2-METHYL-3-OXOSUCCINATE | C9H14O5 |
| PALATINOSE | C12H22O11 |  | RETINYL PALMITATE | C36H60O2 |
| D-PINITOL | C7H14O6 |  | 2-UNDECANONE | C11H22O |
| MALTOSE | C12H22O11 |  | TRANS-CINNAMATE | C9H8O2 |
| TAGATOSE | C6H12O6 |  | GLYCEROL-MYRISTATE | C17H34O4 |
| L-GULONOLACTONE | C6H10O6 |  | OLEATE | C18H34O2 |
| ARABINOSE | C5H10O5 |  | STEARATE | C18H36O2 |
| CELLOBIOSE | C12H22O11 |  | 25-HYDROXYCHOLESTEROL | C27H46O2 |
| PSICOSE | C6H12O6 |  | NERVONATE | C24H46O2 |
| ARABITOL | C5H12O5 |  | DESMOSTEROL | C27H44O |
| LYXOSE | C5H10O5 |  | DEOXYCORTICOSTERONE ACETATE | C23H32O4 |
| VITAMIN D2 | C28H44O |  | OLEOYL-GLYCEROL | C21H40O4 |
| CORTICOSTERONE | C21H30O4 |  | CORTISONE | C21H28O5 |
| LITHOCHOLATE | C24H40O3 |  | URIC ACID | C5H4N4O3 |
| PROTOPORPHYRIN | C34H34N4O4 |  |  |  |
| 4-COUMARATE | C9H8O3 |  |  |  |
| NONANOATE | C9H18O2 |  |  |  |
| ESTRADIOL-17ALPHA | C18H24O2 |  |  |  |
| CAPRYLATE | C8H16O2 |  |  |  |
| URSODEOXYCHOLATE | C24H40O4 |  |  |  |
| PETROSELINATE | C18H34O2 |  |  |  |
| DIPALMITOYLGLYCEROL | C35H68O5 |  |  |  |
| HEPTANOATE | C7H14O2 |  |  |  |
| CORTEXOLONE | C21H30O4 |  |  |  |
| LITHOCHOLYLTAURINE | C26H45NO5S |  |  |  |
| PALMITOLEATE | C16H30O2 |  |  |  |
| MENAQUINONE | C31H40O2 |  |  |  |
| ELAIDATE | C18H34O2 |  |  |  |
| CHENODEOXYCHOLATE | C24H40O4 |  |  |  |
| MYRISTATE | C14H28O2 |  |  |  |
| PALMITATE | C16H32O2 |  |  |  |
| EICOSAPENTAENOATE | C20H30O2 |  |  |  |
| HEPTADECANOATE | C17H34O2 |  |  |  |
| LIOTHYRONINE | C15H12I3NO4 |  |  |  |
| SPHINGANINE | C18H39NO2 |  |  |  |
| LAURATE | C12H24O2 |  |  |  |
| ARACHIDATE | C20H40O2 |  |  |  |
| DEOXYCHOLATE | C24H40O4 |  |  |  |
| KETOLEUCINE | C6H10O3 |  |  |  |
| LINOLEATE | C18H32O2 |  |  |  |
| 5,6 DIMETHYLBENZIMIDAZOLE | C9H10N2 |  |  |  |
| RETINOATE | C20H28O2 |  |  |  |
| SPHINGOMYELIN | C41H83N2O6P |  |  |  |
| 7-DEHYDROCHOLESTEROL | C27H44O |  |  |  |
| BIS(2-ETHYLHEXYL)PHTHALATE | C24H38O4 |  |  |  |
| GAMMA-LINOLENATE | C18H30O2 |  |  |  |
| OMEGA-HYDROXYDODECANOATE | C12H24O3 |  |  |  |
| METHYL JASMONATE | C13H20O3 |  |  |  |
| INDOLE | C8H7N |  |  |  |

**Table S1e.** List of reference metabolites that were included in an in-house library, continued.

| **Formula^1^** | ***m/z*** | **Ion** | **Δm (ppm)** | **t_R_^2^ (min)** | **VIP^3^** | ***P*-value^4^** | **Putative annotation** | **Most specific class^5^** | **Probability** | **Class or subclass^6^** | **Direction^7^** |
| --- | --- | --- | --- | --- | --- | --- | --- | --- | --- | --- | --- |
| C_5_H_5_NO_3_ | 114.019 | [M−H]^−^ | −3.7 | 15.55 | 2.22 | <0.01 | maleamic acid | α-amino acids and derivatives | 0.54 | carboxylic acids and derivatives | ↓ |
| C_13_H_22_N_4_O_8_S_2_ | 427.096 | [M+H]^+^ | 0.39 | 17.29 | 2.13 | 0.03 | *S*-glutathionyl-L-cysteine | γ-glutamyl peptides | 0.99 | - | ↓ |
| C_6_H_13_NO_4_ | 164.092 | [M+H]^+^ | 2.1 | 11.44 | 2.12 | <0.01 | - | - | - | - | ↑ |
| C_7_H_14_O_8_ | 225.061 | [M−H]^−^ | −1.9 | 17.70 | 2.06 | <0.01 | - | quinic acids and derivatives | 0.54 | cyclic alcohols and derivatives | ↑ |
| C_6_H_9_NO_4_S | 190.018 | [M−H]^−^ | −0.79 | 16.63 | 1.99 | <0.01 | - | α-amino acids | 0.62 | amino acids and derivatives | ↓ |
| C_30_H_50_O_2_ | 460.415 | [M+NH_4_]^+^ | 0.96 | 3.12 | 1.86 | <0.01 | - | prenol lipids | 0.80 | lipids and lipid-like molecules | ↑ |
| C_11_H_21_NO_9_ | 310.114 | [M−H]^−^ | −1.5 | 14.73 | 1.86 | <0.01 | - | fatty acyl glycosides of mono- and disaccharides | 0.57 | - | ↑ |
| C_13_H_30_N_3_O_10_PS_2_ | 484.117 | [M+H]^+^ | −2.9 | 16.72 | 1.84 | 0.04 | - | - | - | - | ↓ |
| C_52_H_81_N_7_O_12_ | 498.804 | [M+2H]^2+^ | −0.14 | 3.07 | 1.83 | 0.02 | - | - | - | - | ↑ |
| C_5_H_8_N_8_O_5_ | 261.070 | [M+H]^+^ | 2.7 | 4.60 | 1.83 | 0.03 | - | - | - | - | ↑ |
| C_56_H_111_N_8_O_9_P_3_S | 583.378 | [M+2H]^2+^ | 0.38 | 3.08 | 1.82 | 0.02 | - | - | - | - | ↑ |
| C_6_H_10_O_4_S | 177.022 | [M−H]^−^ | −2.5 | 7.66 | 1.75 | <0.01 | - | short-chain hydroxy acids and derivatives | 0.67 | organic acids and derivatives | ↑ |
| C_25_H_29_N_7_O_5_S_2_ | 286.591 | [M+2H]^2+^ | 1.6 | 15.10 | 1.70 | 0.05 | - | - | - | - | ↑ |
| C_14_H_18_ClN_7_O_5_ | 398.098 | [M−H]^−^ | −1.4 | 15.01 | 1.69 | <0.01 | - | - | - | - | ↑ |
| C_8_H_16_N_2_O_9_ | 283.078 | [M−H]^−^ | −1.8 | 11.80 | 1.67 | <0.01 | - | - | - | - | ↑ |
| C_13_H_29_N_3_O_6_S | 356.185 | [M+H]^+^ | 0.47 | 11.09 | 1.64 | 0.03 | - | amino acids and derivatives | 0.84 | organic acids and derivatives | ↓ |
| C_4_H_11_NO | 90.0915 | [M+H]^+^ | 2.1 | 17.87 | 1.59 | 0.03 | - | - | - | - | ↑ |
| C_13_H_24_N_2_O_7_S | 351.122 | [M−H]^−^ | −2.0 | 8.75 | 1.58 | 0.02 | - | β-amino acids and derivatives | 0.76 | carboxylic acids and derivatives | ↓ |
| C_34_H_44_N_5_O_3_P | 602.323 | [M+H]^+^ | −4.1 | 3.87 | 1.54 | 0.02 | - | amino acids and derivatives | 0.80 | carboxylic acids and derivatives | ↓ |
| C_11_H_16_N_2_O_2_ | 209.129 | [M+H]^+^ | 1.2 | 8.72 | 1.53 | 0.05 | - | α-amino acids and derivatives | 0.86 | amino acids and derivatives | ↑ |
| C_11_H_19_NO_9_ | 310.114 | [M+H]^+^ | 0.17 | 16.89 | 1.52 | 0.05 | *N*-acetylneuraminic acid | *N*-acylneuraminic acids | 0.81 | sugar acids and derivatives | ↑ |

^1^ Neutral formula from Compound Discoverer software

^2^ t_R_: retention time

^3^ Variable importance in projection score from OPLS-DA model

^4^ *P*-value from pairwise *t*-test

^5^ ClassyFire most specific compound class from CANOPUS

^6^ClassyFire level 5, subclass, class or superclass with probability ≥0.85

^7^Relative change in concentration following exposure with nicotine, i.e., increase (↑) or decrease (↓)

**Table S2.** Complete list of differential metabolites in THP-1 cell extracts (1 h exposure) for which no reference standards were available. Inclusion criteria were VIP≥1.5, *P*≤0.05 and available MS/MS data.

| **Formula^1^** | ***m/z*** | **Ion** | **Δm (ppm)** | **t_R_^2^ (min)** | **VIP^3^** | ***P*-value^4^** | **Putative annotation** | **Most specific class^5^** | **Probability** | **Class or subclass^6^** | **Direction^7^** |
| --- | --- | --- | --- | --- | --- | --- | --- | --- | --- | --- | --- |
| C_10_H_17_N_3_O_6_ | 276.119 | [M+H]^+^ | 0.11 | 15.64 | 2.22 | 0.04 | - | - | - | - | ↓ |
| C_7_H_15_N_3_O_2_ | 174.124 | [M+H]^+^ | 1.9 | 23.45 | 2.18 | 0.04 | - | - | - | - | ↓ |
| C_9_H_11_NO_3_ | 182.081 | [M+H]^+^ | 1.4 | 8.10 | 2.18 | <0.01 | - | aryl alkyl ketones | 0.96 | - | ↑ |
| C_6_H_12_O_7_ | 195.051 | [M−H]^−^ | −2.7 | 14.44 | 1.89 | 0.03 | - | - | - | - | ↓ |
| C_8_H_9_NO_2_ | 197.104 | [M+NH_4_]^+^ | 2.2 | 13.58 | 1.76 | 0.04 | - | - | - | - | ↓ |
| C_9_H_15_N_3_O_2_S | 230.096 | [M+H]^+^ | 1.1 | 15.12 | 1.71 | 0.04 | - | - | - | - | ↑ |
| C_3_H_3_N_7_O_2_ | 168.027 | [M−H]^−^ | −1.1 | 15.32 | 1.69 | 0.04 | - | - | - | - | ↓ |
| C_6_H_9_N_3_O_2_ | 154.062 | [M−H]^−^ | 0.97 | 15.64 | 1.56 | 0.05 | - | - | - | - | ↓ |
| C_9_H_9_NO | 146.061 | [M−H]^−^ | −3.5 | 4.65 | 1.53 | 0.04 | - | - | - | - | ↓ |

^1^ Neutral formula from Compound Discoverer software

^2^ t_R_: retention time

^3^ Variable importance in projection score from OPLS-DA model

^4^ *P*-value from pairwise *t*-test

^5^ ClassyFire most specific compound class from CANOPUS

^6^ClassyFire level 5, subclass, class or superclass with probability ≥0.85

^7^Relative change in concentration following exposure with nicotine, i.e., increase (↑) or decrease (↓)

**Table S3.** Complete list of differential metabolites in THP-1 medium extracts (1 h exposure) for which no reference standards were available. Inclusion criteria were VIP≥1.5, *P*≤0.05 and available MS/MS data.

| **Formula^1^** | ***m/z*** | **Ion** | **Δm (ppm)** | **t_R_^2^ (min)** | **VIP^3^** | ***P*-value^4^** | **Putative annotation** | **Most specific class^5^** | **Probability** | **Class or subclass^6^** | **Direction^7^** |
| --- | --- | --- | --- | --- | --- | --- | --- | --- | --- | --- | --- |
| C_11_H_13_N_3_ | 188.118 | [M+H]^+^ | 1.0 | 3.93 | 2.62 | <0.01 | - | - | - | - | ↑ |
| C_10_H_11_NO_2_ | 178.087 | [M+H]^+^ | 1.8 | 11.43 | 2.33 | 0.01 | - | - | - | - | ↓ |
| C_9_H_9_N | 132.081 | [M+H]^+^ | 1.0 | 4.79 | 2.31 | <0.01 | methylindole | indoles | 0.77 | organoheterocyclic compounds | ↑ |
| C_5_H_10_N_2_O_2_ | 147.077 | [M+H]^+^ | 1.9 | 11.44 | 2.27 | <0.01 | ureidoisobutyric acid | α-amino acids | 0.95 |  | ↑ |
| C_4_H_10_N_2_O_2_S | 151.054 | [M+H]^+^ | 2.0 | 13.71 | 2.23 | 0.03 | - | monoalkylamines | 0.62 | organonitrogen compounds | ↑ |
| C_6_H_6_O_4_S | 172.991 | [M−H]^−^ | −3.1 | 4.85 | 2.16 | <0.01 | - | phenylsulfates | 0.68 | organic sulfuric acids and derivatives | ↓ |
| C_4_H_7_NO_2_S | 134.027 | [M+H]^+^ | −0.11 | 8.75 | 2.10 | 0.03 | - | α-amino acids | 0.59 | amino acids and derivatives | ↑ |
| C_9_H_13_N_3_O_4_ | 228.098 | [M+H]^+^ | 1.3 | 10.34 | 2.05 | <0.01 | deoxycytidine | pyrimidine 2'-deoxyribonucleosides | >0.99 |  | ↓ |
| C_40_H_78_NO_8_P | 732.555 | [M+H]^+^ | 1.3 | 3.25 | 1.99 | <0.01 | PE-Me(16:0/18:1) | phosphatidylcholines | 0.99 |  | ↓ |
| C_5_H_4_N_4_O_3_ | 167.021 | [M−H]^−^ | −2.7 | 13.76 | 1.92 | 0.02 | uric acid | xanthines | 0.92 |  | ↑ |
| C_5_H_11_ClN_2_O_3_ | 181.038 | [M−H]^−^ | −2.7 | 15.35 | 1.82 | 0.03 | - | α-amino acids | 0.53 | amino acids and derivatives | ↑ |
| C_9_H_7_N | 130.065 | [M+H]^+^ | 1.3 | 4.75 | 1.77 | 0.02 | - | benzenoids | 0.97 |  | ↑ |
| C_10_H_20_N_2_O_5_ | 249.145 | [M+H]^+^ | 1.1 | 7.88 | 1.74 | <0.01 | - | α-amino acids and derivatives | 0.77 | amino acids and derivatives | ↑ |
| C_10_H_20_O_11_ | 315.093 | [M−H]^−^ | −1.5 | 13.69 | 1.66 | <0.01 | - | fatty acyl glycosides of mono- and disaccharides | 0.51 | - | ↑ |
| C_10_H_13_ClN_2_ | 197.084 | [M+H]^+^ | 1.2 | 4.12 | 1.62 | 0.01 | - | - | - | - | ↑ |
| C_9_H_9_NO_3_^8^ | 180.066 | [M+H]^+^ | 0.75 | 6.88 | 1.59 | 0.02 | - | aryl alkylketones | 0.79 | carbonyl compounds | ↑ |
| C_9_H_11_NO_3_^9^ | 182.081 | [M+H]^+^ | 1.4 | 8.10 | 1.57 | 0.01 | - | aryl alkylketones | 0.79 | carbonyl compounds | ↑ |
| C_6_H_9_N_3_O_2_ | 156.077 | [M+H]^+^ | 1.4 | 15.24 | 1.56 | 0.03 | - | - | - | - | ↑ |

^1^ Neutral formula from Compound Discoverer software

^2^ t_R_: retention time

^3^ Variable importance in projection score from OPLS-DA model

^4^ *P*-value from pairwise *t*-test

^5^ ClassyFire most specific compound class from CANOPUS

^6^ClassyFire level 5, subclass, class or superclass with probability ≥0.85

^7^Relative change in concentration following exposure with nicotine, i.e., increase (↑) or decrease (↓)

^8^ Putatively annotated as biotransformation product of nicotine, 4-oxo-4-(3-pyridyl)-butanoic acid

^9^ Putatively annotated as biotransformation product of nicotine, 4-hydroxy-4-(3-pyridyl)-butanoic acid

**Table S4.** Complete list of differential metabolites in THP-1 medium extracts (4 h exposure) for which no reference standards were available. Inclusion criteria were VIP≥1.5, *P*≤0.05 and available MS/MS data.

| **Amino acid or amino acid combination** | **Cell extract, exposed vs non-exposed T1** | **Cell extract, exposed vs non-exposed T4** | **Medium, exposed vs non-exposed T1** | **Medium, exposed vs non-exposed T4** | **Cell extract, exposed T1 vs T4** | **Cell extract, non-exposed T1 vs T4** |
| --- | --- | --- | --- | --- | --- | --- |
| Ala | 0.81 | 0.96 | 0.15 | 0.13 | 0.63 | 0.49 |
| Arg | 0.58 | 0.83 | **0.05** | 0.11 | 0.55 | 0.43 |
| Asn | 0.82 | 0.87 | **0.02** | **<0.01** | 0.67 | 0.38 |
| Asp | 0.62 | 0.82 | **0.04** | **<0.01** | 0.59 | 0.38 |
| Cit | 0.36 | 0.79 | **0.04** | **0.03** | 0.40 | 0.45 |
| Glu | 0.66 | 0.80 | **0.02** | **<0.01** | 0.66 | 0.42 |
| Gly | 0.71 | 0.60 | 0.12 | 0.92 | 0.71 | 0.95 |
| Lys | 0.39 | 0.74 | **0.04** | 0.07 | 0.41 | 0.40 |
| Met | 0.54 | 0.81 | 0.06 | **0.01** | 0.53 | 0.39 |
| Orn | 0.31 | 0.68 | 0.10 | 0.19 | 0.41 | 0.43 |
| Phe | 0.12 | 0.76 | 0.44 | 0.62 | 0.35 | 0.29 |
| Pro | 0.85 | 0.92 | 0.11 | 0.11 | 0.69 | 0.41 |
| Ser | 0.37 | 0.85 | 0.05 | **<0.01** | 0.32 | 0.21 |
| Thr | 0.55 | 0.87 | **0.04** | **0.04** | 0.51 | 0.42 |
| Tyr | 0.55 | 0.79 | 0.07 | **0.04** | 0.55 | 0.40 |
| SDMA | 0.34 | 0.15 | **0.02** | 0.74 | 0.30 | 0.44 |
| t4-OH-Pro | 0.65 | 0.85 | 0.14 | 0.05 | 0.60 | 0.42 |
| Taurine | 0.85 | 0.74 | 0.08 | 0.45 | 0.61 | 0.45 |
| Asn / Asp | 0.77 | 0.91 | 0.07 | **0.02** | 0.75 | 0.38 |
| Pro / Phe | 0.32 | 0.99 | **0.03** | 0.32 | 0.26 | 0.59 |
| Arg / (Orn + Cit) | 0.05 | 0.83 | 0.23 | 0.19 | 0.06 | 0.43 |
| Gly / Ser | 1.00 | 0.73 | 0.70 | 0.67 | 0.99 | 0.89 |
| Met / Phe | 0.27 | 0.85 | **0.03** | 0.24 | 0.25 | 0.53 |
| Tyr / Phe | 0.27 | 0.82 | **0.02** | 0.23 | 0.26 | 0.54 |
| (Ala + Arg + Asn + Asp + Cys + Gln + Glu + Gly + Pro + Ser + Tyr) / (His + Ile + Leu + Lys + Met + Phe + Thr + Trp + Val) | **0.03** | 0.96 | **0.02** | 0.31 | **0.03** | 0.76 |
| Pro / Cit | 0.06 | 0.85 | **<0.001** | 0.07 | 0.05 | 0.46 |
| Ala + Arg + Asn + Asp + Cys + Gln + Glu + Gly + His + Ile + Leu + Lys + Met + Phe + Pro + Ser + Thr + Trp + Tyr + Val | 0.74 | 0.88 | 0.08 | **0.01** | 0.64 | 0.41 |
| Phe + Trp + Tyr | 0.56 | 0.79 | 0.07 | 0.05 | 0.55 | 0.40 |
| His + Ile + Leu + Lys + Met + Phe + Thr + Trp + Val | 0.47 | 0.81 | **0.04** | **0.04** | 0.47 | 0.40 |
| Ala + Arg + Asn + Asp + Cys + Gln + Glu + Gly + Pro + Ser + Tyr | 0.77 | 0.89 | 0.08 | **0.02** | 0.66 | 0.41 |

**Table S5a.** *P*-values from paired *t*-tests of amino acid levels, selected amino acid ratios, and sums of amino acids from comparisons between exposed vs. control groups, and between time points T1 (1 h exposure) and T4 (4 h exposure). Effects on specific amino acid ratios and sums may give insight into biochemical events that could be related to nicotine exposures. Statistically significant (*P*≤0.05) outcomes are emphasised.

| **Amino acid or amino acid combination** | **Cell extract, exposed vs non-exposed T1** | **Cell extract, exposed vs non-exposed T4** | **Medium, exposed vs non-exposed T1** | **Medium, exposed vs non-exposed T4** | **Cell extract, exposed T1 vs T4** | **Cell extract, non-exposed T1 vs T4** |
| --- | --- | --- | --- | --- | --- | --- |
| Ala + Arg + Asn + Asp + Cys + Gln + Glu + Gly + His + Met + Pro + Ser + Thr + Val | 0.76 | 0.89 | 0.08 | **0.02** | 0.65 | 0.41 |
| Cit / Orn | 0.45 | 0.68 | 0.20 | 0.68 | 0.51 | 0.40 |
| Cit / Phe | 0.18 | 0.92 | **0.01** | 0.23 | 0.18 | 0.98 |
| Cit / Arg | 0.33 | 0.60 | **0.03** | 0.16 | 0.38 | 0.40 |
| Orn / Arg | **0.02** | 0.69 | 0.43 | 0.33 | **0.01** | 0.43 |
| Arg/Orn | **0.01** | 0.85 | 0.43 | 0.30 | **<0.01** | 0.44 |
| Orn / Cit | 0.70 | 0.64 | 0.20 | 0.67 | 0.81 | 0.40 |
| SDMA^1^ / Arg | 0.42 | 0.74 | 0.77 | 0.77 | 0.65 | 0.38 |
| Arg / (Orn + Cit) | 0.05 | 0.83 | 0.23 | 0.19 | 0.06 | 0.43 |

^1^ Symmetric dimethylarginine

**Table S5b.** *P*-values from paired *t*-tests of amino acid levels, selected amino acid ratios, and sums of amino acids from comparisons between exposed vs. control groups, and between time points T1 (1 h exposure) and T4 (4 h exposure). Effects on specific amino acid ratios and sums may give insight into biochemical events that could be related to nicotine exposures. Statistically significant (*P*≤0.05) outcomes are emphasised.

| **Amino acid or group of amino acids** | **Explanation** | **Diagnostic relevance** |
| --- | --- | --- |
| Asn / Asp | Ratio of asparagine to aspartate | Indicator of asparagine synthetase activity |
| Pro / Phe | Ratio of proline to phenylalanine | Altered degradation of the amino acids leucine, isoleucine, and valine |
| Fischer ratio | Ratio of branched-chain amino acids to aromatic amino acids | Predictor of cardiac events and liver damage |
| Gly / Ser | Ratio of glycine to serine | Indicator of glycine synthesis |
| Met / Phe | Ratio of methionine to phenylalanine | Indicator of folate metabolism |
| Global arginine bioavailability ratio | Ratio of arginine to sum of ornithine and citrulline | Associated with endothelial dysfunction and increased risk of cardiovascular mortality |
| [Ala + Arg + Asn + Asp + Cys + Gln + Glu + Gly + Pro + Ser + Tyr]/[His + Ile + Leu + Lys + Met + Phe + Thr + Trp + Val] | Fraction of non-essential amino acids relative to essential amino acids | Amino acid metabolism |
| Pro/Cit | Ratio of proline to citrulline | Activity of ornithine aminotransferase and pyrroline-5-carboxylate reductase and an indirect indicator of the activity of arginase to nitric oxide synthase (NOS) |
| Ala + Arg + Asn + Asp + Cys + Gln + Glu + Gly + His + Ile + Leu + Lys + Met + Phe + Pro + Ser + Thr + Trp + Tyr + Val | Sum of amino acids | AAs are the basic units of proteins and contain an amino group and a carboxylic group |
| Phe + Trp + Tyr | Sum of aromatic amino acids | Indicator of the development of insulin resistance and nutritional status |
| Ile + Leu + Val | Sum of branched chain amino acids | An inndicator for short-term metabolic control |
| His + Ile + Leu + Lys + Met + Phe + Thr + Trp + Val | Sum of essential amino acids | An inndicator of the nutritional status |
| Ala + Arg + Asn + Asp + Cys + Gln + Glu + Gly + Pro + Ser + Tyr | Sum of non-essential amino acids | An inndicator for protein turn-over |
| Ala + Arg + Asn + Asp + Cys + Gln + Glu + Gly + His + Met + Pro + Ser + Thr + Val | Sum of solely glucogenic AAs | An indicator for gluconeogenic activity and endogenous glucose production |
| Val / Phe | Ratio of valine to phenylalanine | Activity of valine transaminase |
| ADMA / Arg | Ratio of asymmetrically dimethylated arginine to total unmodified arginine | Inhibition of NO synthase, associated with endothelial dysfunction and cardiovascular risk in general |
| t4-OH-Pro / Pro | Ratio of proline to hydroxyproline | Proline hydroxylation linked to the degradation of connective tissue |
| Cit / Orn | Ratio of citrulline to ornithine | Activity of ornithine carbamoylphosphate transferase |
| Cit / Arg | Ratio of citrulline to arginine | Activity of nitric oxide synthase (NOS) |
| Orn / Cit | Ratio of ornithine to citrulline | Urea cycle disorder that leads to the accumulation of ammonia in blood |
| (Ser + Gly + Ala) / H1 | Ratio of serine, glycine, and alanine to hexose | This ratio is partly affected by the activity of the glycolysis-related enzyme pyruvate kinase |
| (ADMA + SDMA) / Arg | Sum of asymmetrical and symmetrical arginine methylation | An indicator of protein arginine methyl transferase activity |
| ADMA + SDMA | Sum of asymmetric dimethylarginine and symmetric dimethylarginine | Methylarginines as predictor of renal and cardiovascular outcome |
| SDMA / Arg | Ratio of symmetric dimethylarginine to unmodified arginine | The activity of type II protein arginine methyltransferases |

**Table S6a.** Explanations for the diagnostic relevance of changes in amino acid levels, amino acid ratios and sums of amino acids in human settings.

| **Polyamines** | **Explanation** | **Diagnostic relevance** |
| --- | --- | --- |
| Spermidine / putrescine | Spermidine / putrescine | An indicator of spermidine synthase activity converting the polyamine putrescine to spermidine |
| Spermine / spermidine | Spermine / spermidine | An indicator of spermine synthase activity converting the polyamine spermidine to spermine |
| Putrescine + spermidine + spermine | Sum of polyamines | Polyamines are polycations that interact with negatively charged molecules such as DNA, RNA, and proteins: involved in stress resistance |

**Table S6b.** Explanations for the diagnostic relevance of changes in polyamine ratios in human settings.
